# Supplementary material for: FLI1 and FRA1 transcription factors drive the transcriptional regulatory networks characterizing muscle invasive bladder cancer
Source: Commun Biol. 2023 Feb 20;6:199. doi: 10.1038/s42003-023-04561-3 (PMC9941102; doi:10.1038/s42003-023-04561-3)
Supplement: Supplementary file 1 — Supplementary Information [file 42003_2023_4561_MOESM1_ESM.pdf]

Supplementary Information for

FLI1 and FRA1 transcription factors drive the transcriptional regulatory networks characterizing muscle invasive bladder cancer

Perihan Yagmur Guneri-Sozeri[1,2*], Gülden Özden-Yılmaz[1*], Asli Kisim[3], Ece Cakiroglu[1,2],Aleyna Eray[1,2], Hamdiye Uzuner[1,2], Gökhan Karakülah[1,2], Devrim Pesen Okvur[3], Serif Senturk[1,2], Serap Erkek-Ozhan[1]

[1]Izmir Biomedicine and Genome Center, Inciralti 35340, Izmir, Turkey

[2]Dokuz Eylül University Izmir International Biomedicine and Genome Institute, Inciralti

35340, Izmir, Turkey

[3] Izmir Institute of Technology, Urla, 35430, Izmir, Turkey

Correspondence and requests for materials should be addressed to Serap Erkek-Ozhan:

(serap.erkek@ibg.edu.tr)

Supplementary Figure 1. Example of a common enhancer.

Supplementary Figure 2. Distribution of the gene expression values in NMIBC and MIBC cell lines.

Supplementary Figure 3. Comparison of the expression of MIBC and NMIBC target genes in cell lines.

Supplementary Figure 4. Transcription factor motif enrichment analysis in MIBC cell lines.

Supplementary Figure 5. Transcription factor motif enrichment analysis in NMIBC cell lines.

Supplementary Figure 6. Expression level of transcription factors in primary MIBC and NMIBC tissue samples.

Supplementary Figure 7. Co-regulation of FRA1 and FLI1.

Supplementary Figure 8. Motif enrichment analysis at FRA1 and FLI1 ChIP-seq peaks.

Supplementary Figure 9. H3K27ac occupancy profiles of FRA1 and FLI1.

Supplementary Figure 10. Expression level of EMT-related genes after FRA1 and FLI1 knockdown.

Supplementary Figure 11. Association of *MAP4K4* expression with metastatic potential of MIBC.

Supplementary Figure 12. Regulatory hubs implicated in regulation of EMT-related genes.

Supplementary Figure 13. ChIP-seq signal of SWI/SNF complex components at MIBC enhancers regulating MAP4K4 and FLOT1.

Supplementary Figure 14. Knockdown efficiency of FLI1 and FRA1 in T24 cells used in IC-CHIP and differentiation of C2C12 cells and IC-CHIP assay in the presence and absence of FBS.

Supplementary Figure 15. Knockdown efficiency of FLI1 and FRA1 in 5637 cells and comparison of the invasive capacity of 5637 cells into cell-free matrigel in the presence of FBS and muscle microenvironment.

Supplementary Figure 16. Overexpression of FRA1 and FLI1 transcription factors in RT112 cell line.

Supplementary Figure 17. Expression of key MIBC genes in T2 vs T3-T4 stage MIBC patients.

Supplementary Figure 18. Original Western Blot images displayed in Figure 4c-d, Figure S14a, Figure S15a and Figure S16a.

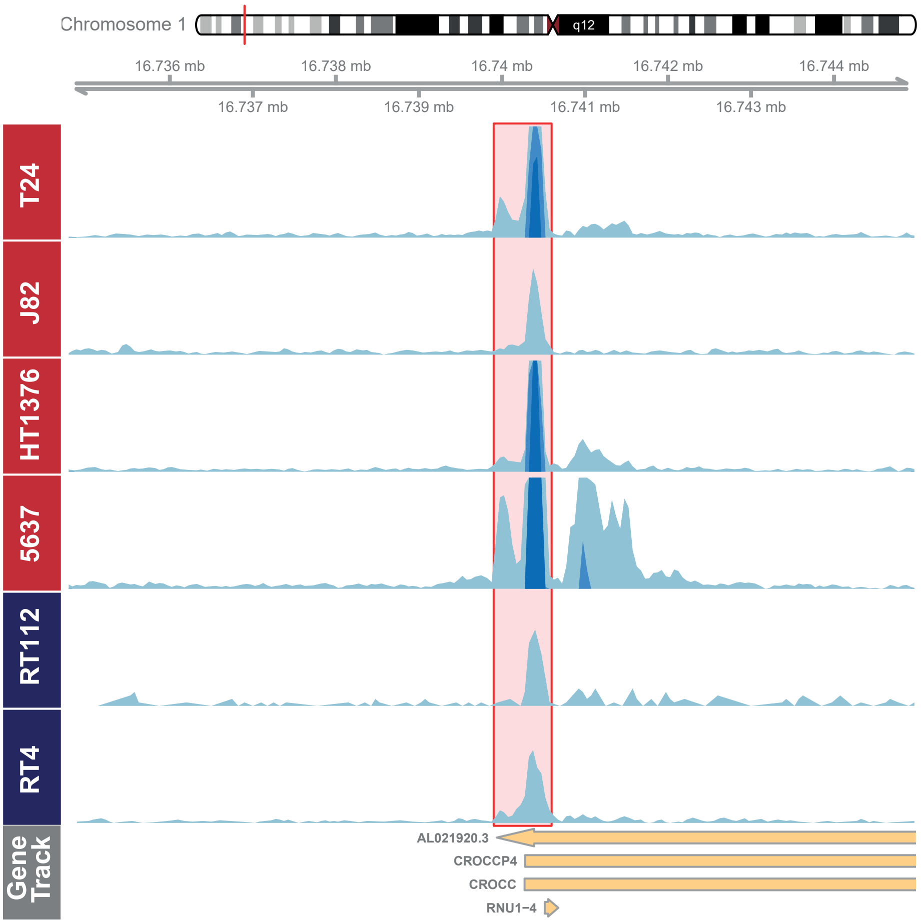

Supplementary Figure 1. Example of a common enhancer. Snapshot visualizes H3K27ac signal at exemplary specific enhancer for NMIBC and MIBC cell lines. Scale of the snapshot is adjusted to 50.

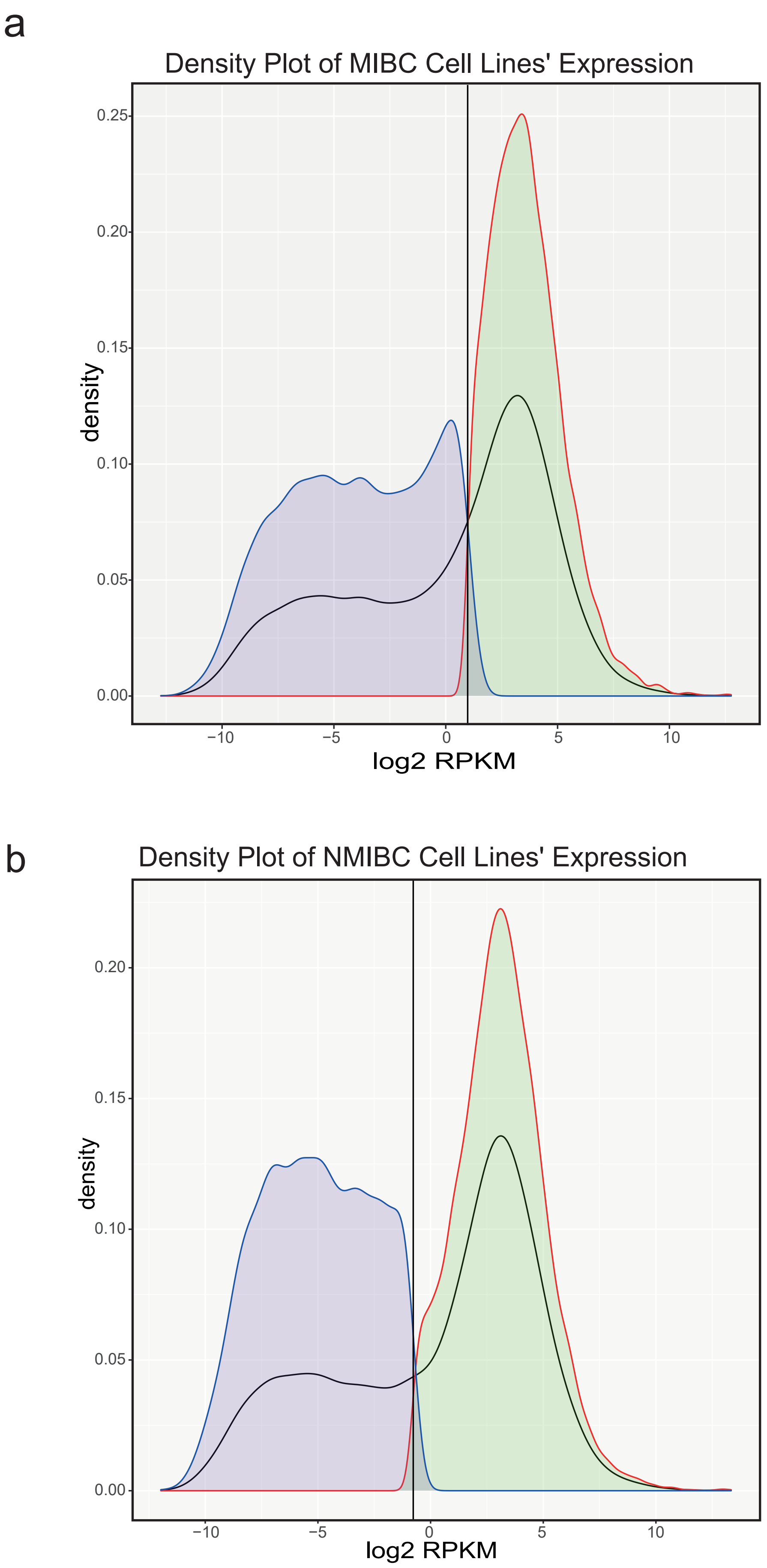

Supplementary Figure 2. Distribution of the gene expression values in NMIBC and MIBC cell lines. (a-b) Density plots displaying the average gene expression values in MIBC (a) and NMIBC (b) cell lines. Expressed genes were shown in green and not-expressed genes are shown in purple (using “G=3” option and p-value <0.05).

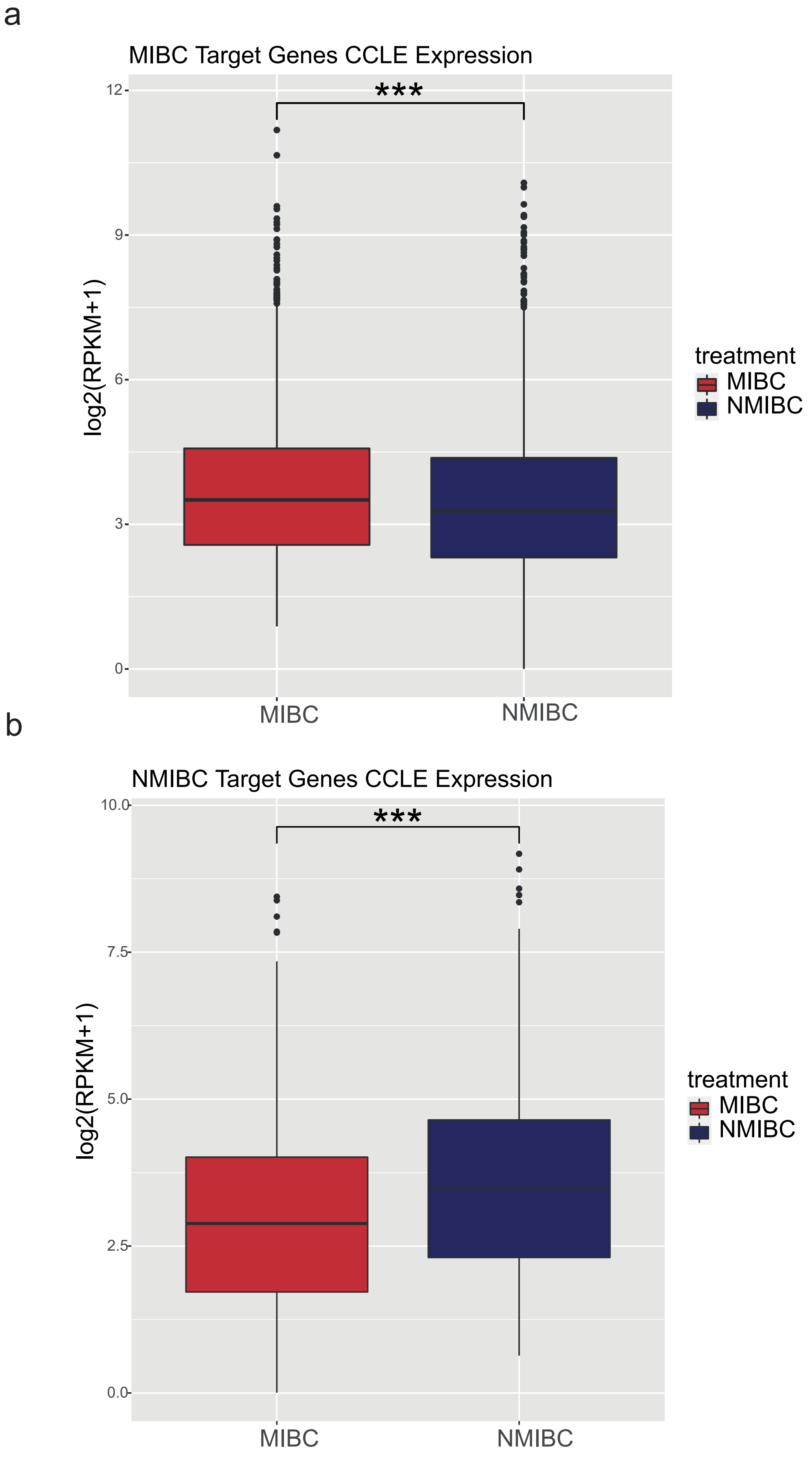

Supplementary Figure 3. Comparison of the expression of MIBC and NMIBC target genes in cell lines. Boxplots showing the expression of MIBC (a) and NMIBC (b) target genes across the NMIBC (n=2) and MIBC cell lines (n=4). *** p value < 0.000001

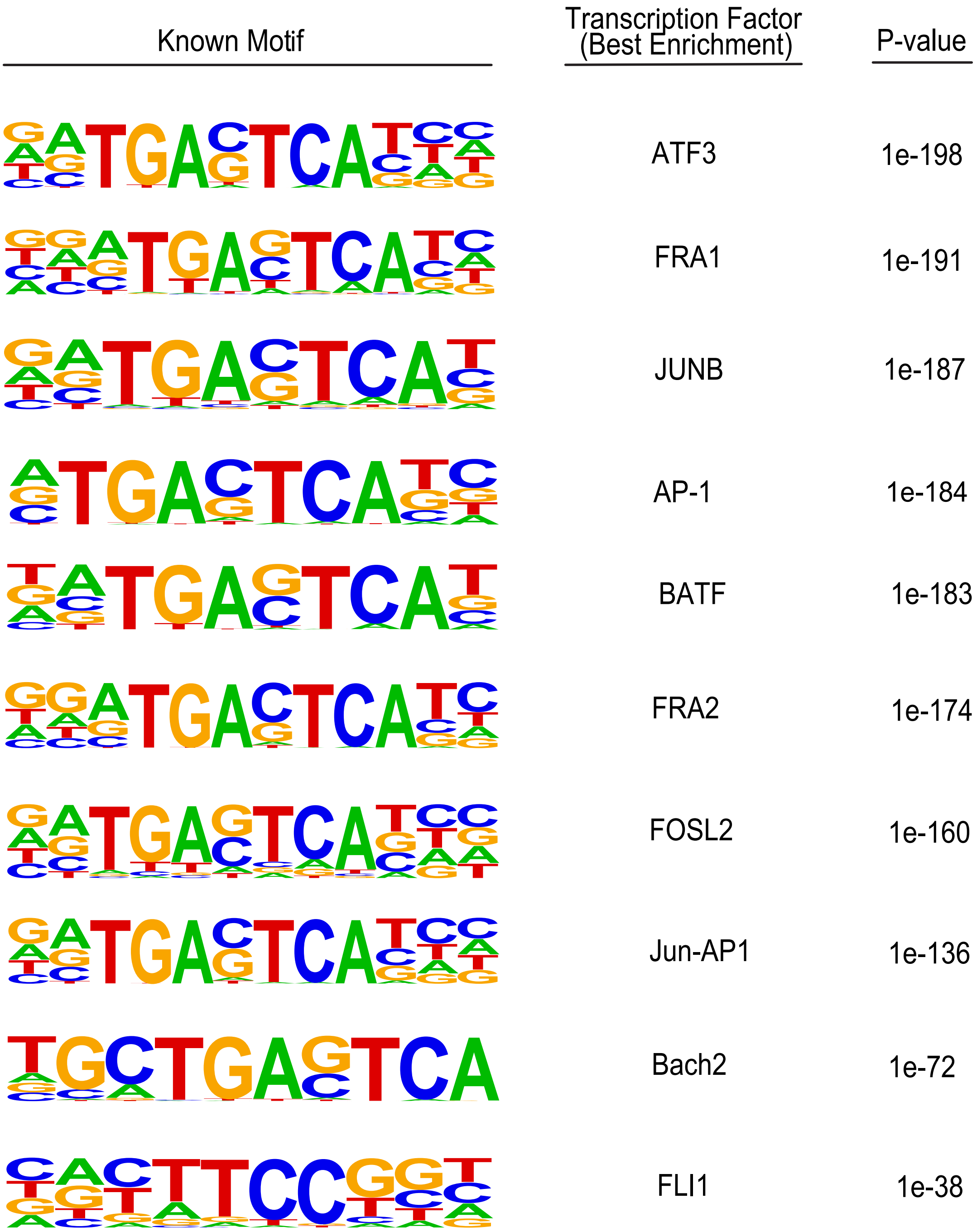

Supplementary Figure 4.Transcription factor motif enrichment analysis in MIBC cell lines. Image shows the top 10 transcription factor motifs enriched at MIBC enhancers.

Known Motif	Transcription Factor (Best Enrichment)	P-value
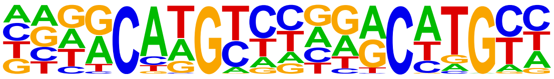	p63	1e-12
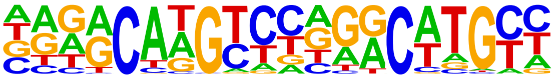	p73	1e-7
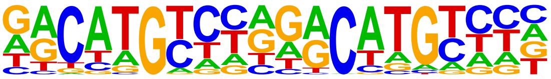	p53	1e-5
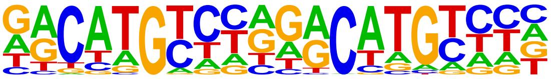	p53	1e-5
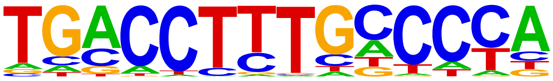	PPARG	1e-4
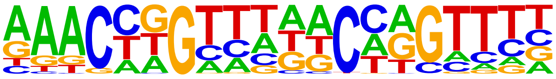	GRHL2	1e-4
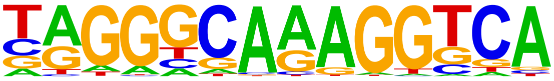	RXR	1e-3
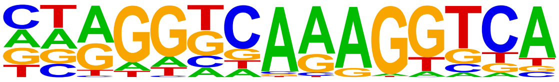	PPARA	1e-3
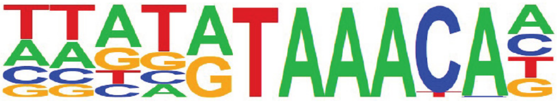	FOXF1	1e-2
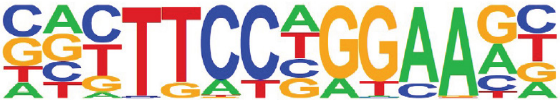	STAT3	1e-2

Supplementary Figure 5.Transcription factor motif enrichment analysis in NMIBC cell lines. Image shows the top 10 transcription factor motifs enriched at NMIBC enhancers.

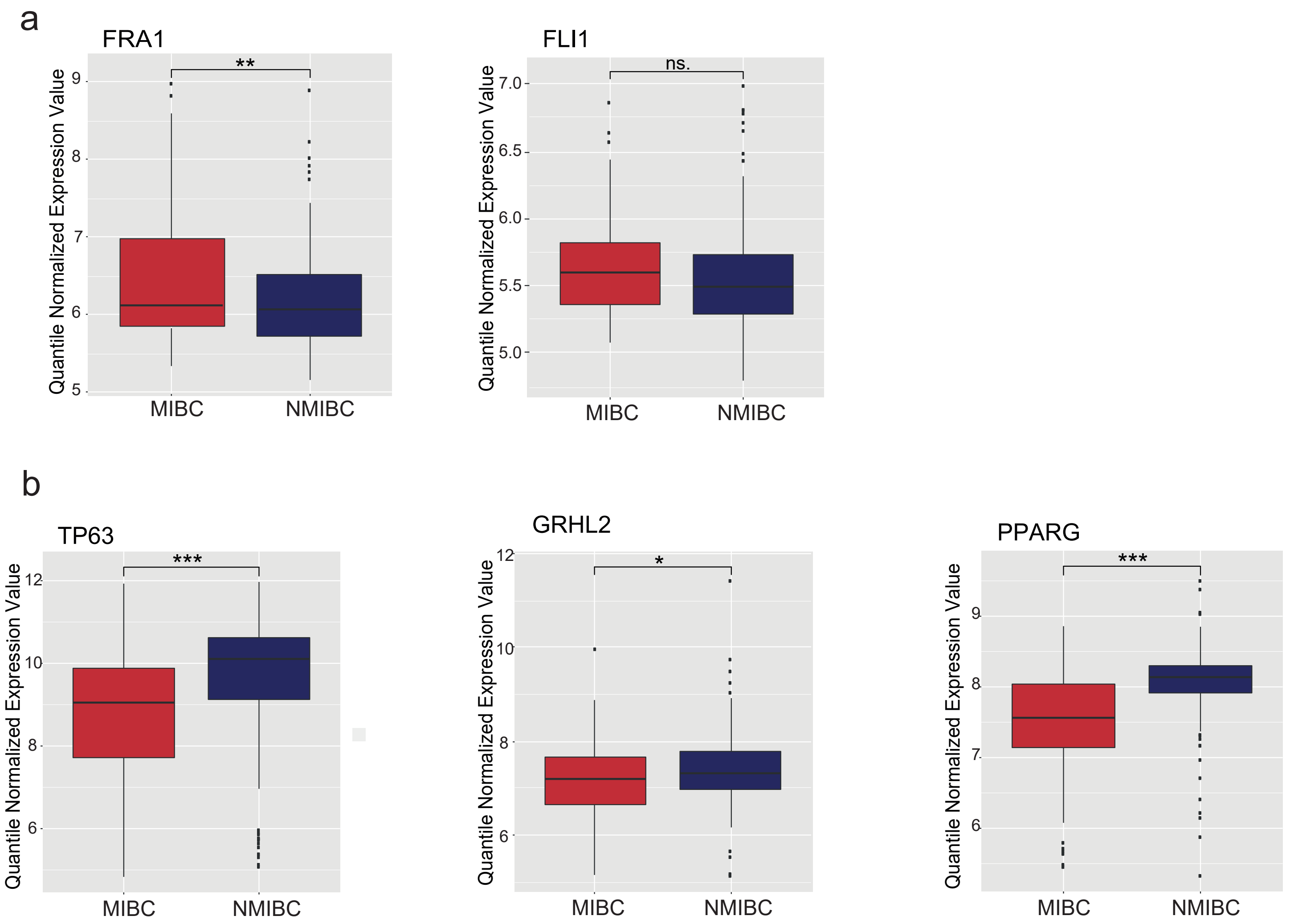

Supplementary Figure 6. Expression level of transcription factors in primary MIBC and NMIBC tissue samples. (a-b) Boxplots display the expression level of FRA1 and FLI1 (b) and the expression of TP63, GRHL2 and PPARG across the NMIBC (n=213) and MIBC primary tissues (n=93).
* p value < 0.05, ** p value < 0.01, *** p value < 0.0001, ns = not significant

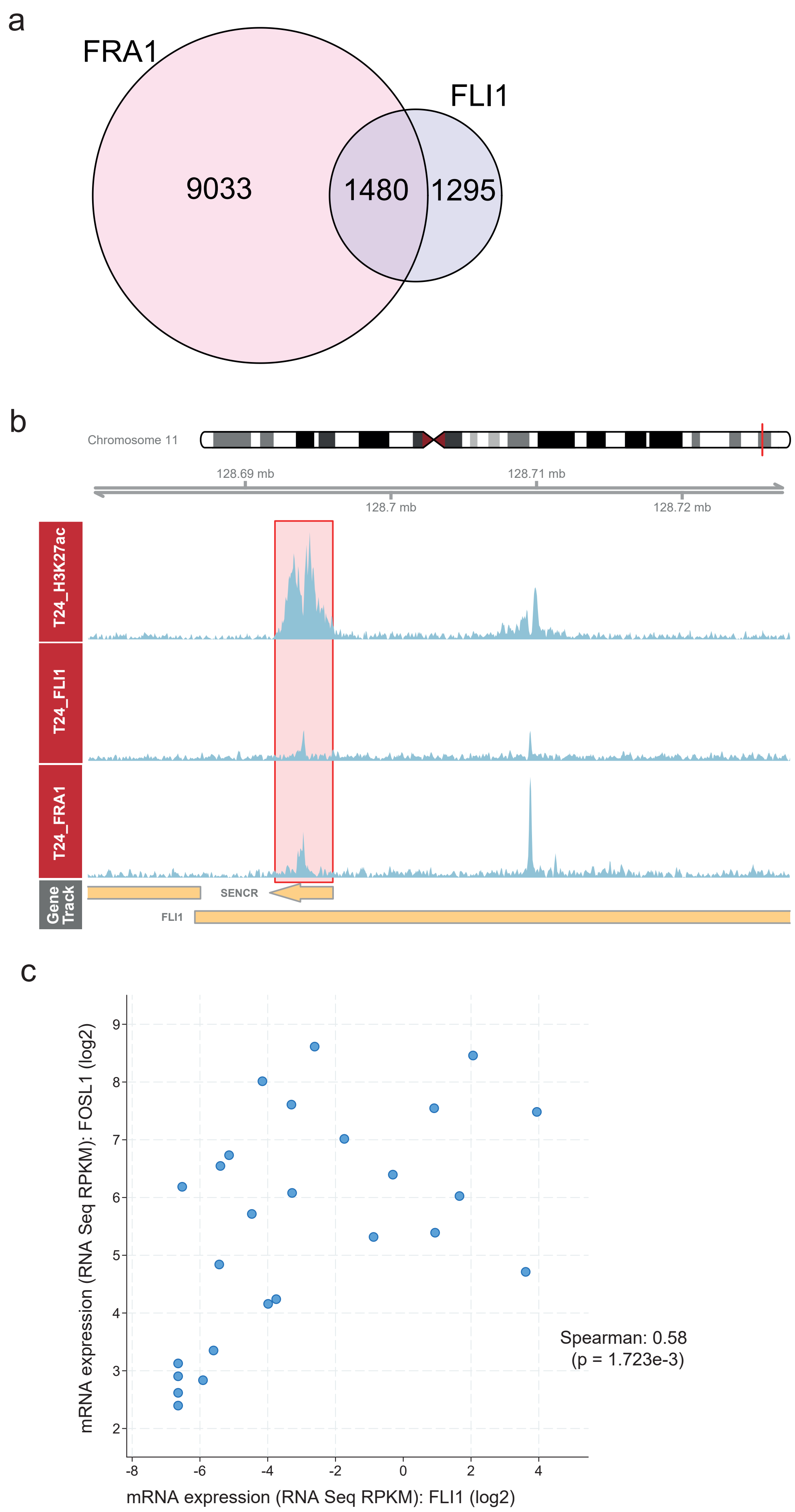

Supplementary Figure 7. Co-regulation of FRA1 and FLI1. (a) Venn diagram showing the overlap between FRA1 and FLI1 peaks in T24 cell line. (b) Snapshot showing H3K27ac, FRA1 and FLI1 signal in T24 cell line at *FLI1* locus. Region specified by red rectangle shows the enhancer regulating FLI1.Scale of the snapshot is adjusted to 25. (c) Scatter plot showing co-expression status for FLI1 and FRA1.

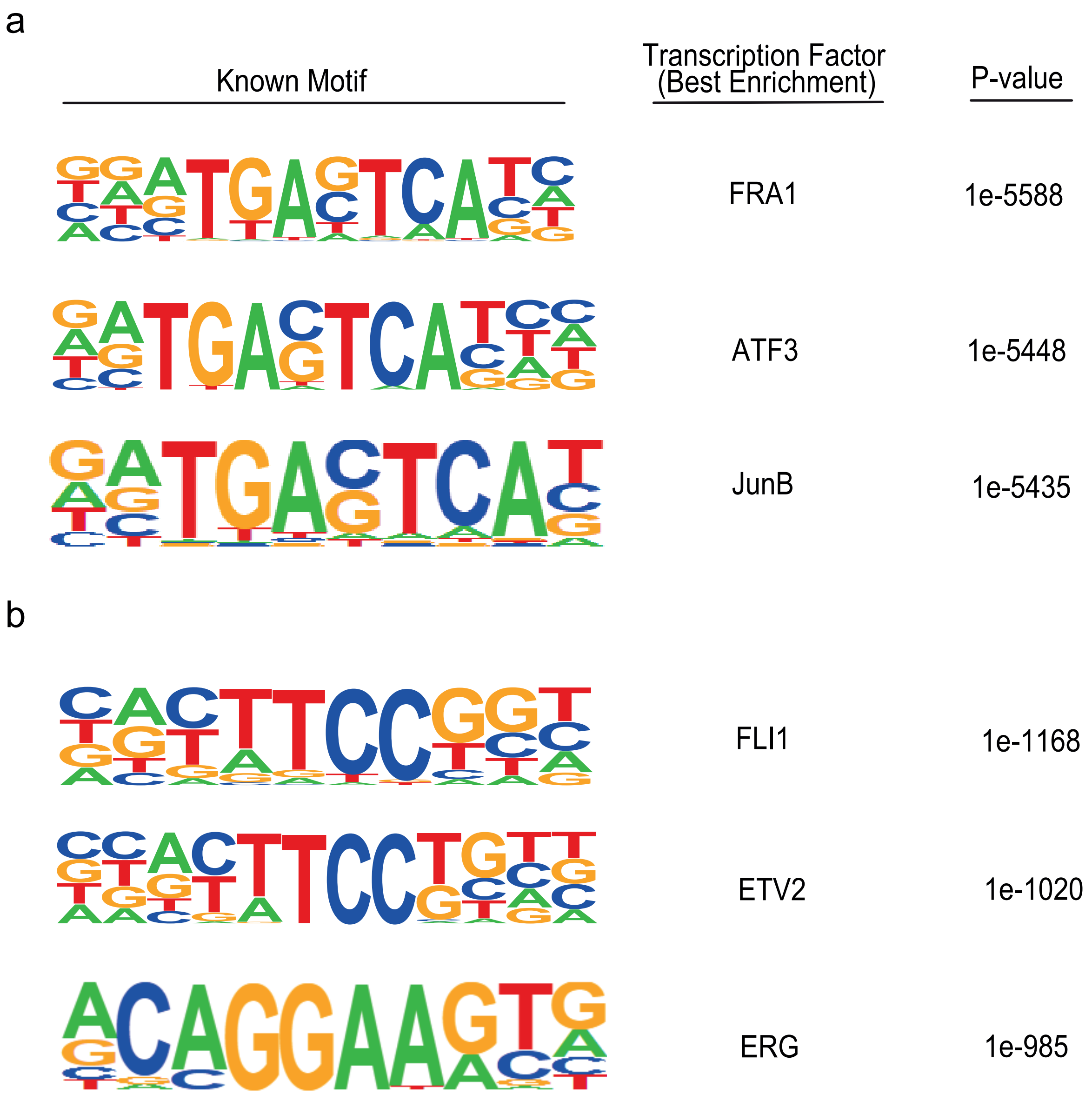

Supplementary Figure 8. Motif enrichment analysis at FRA1 and FLI1 ChIP-seq peaks.
(a-b)Top three transcription factor motifs enriched for FRA1 (a) and FLI1 (b) ChIP-seq data.

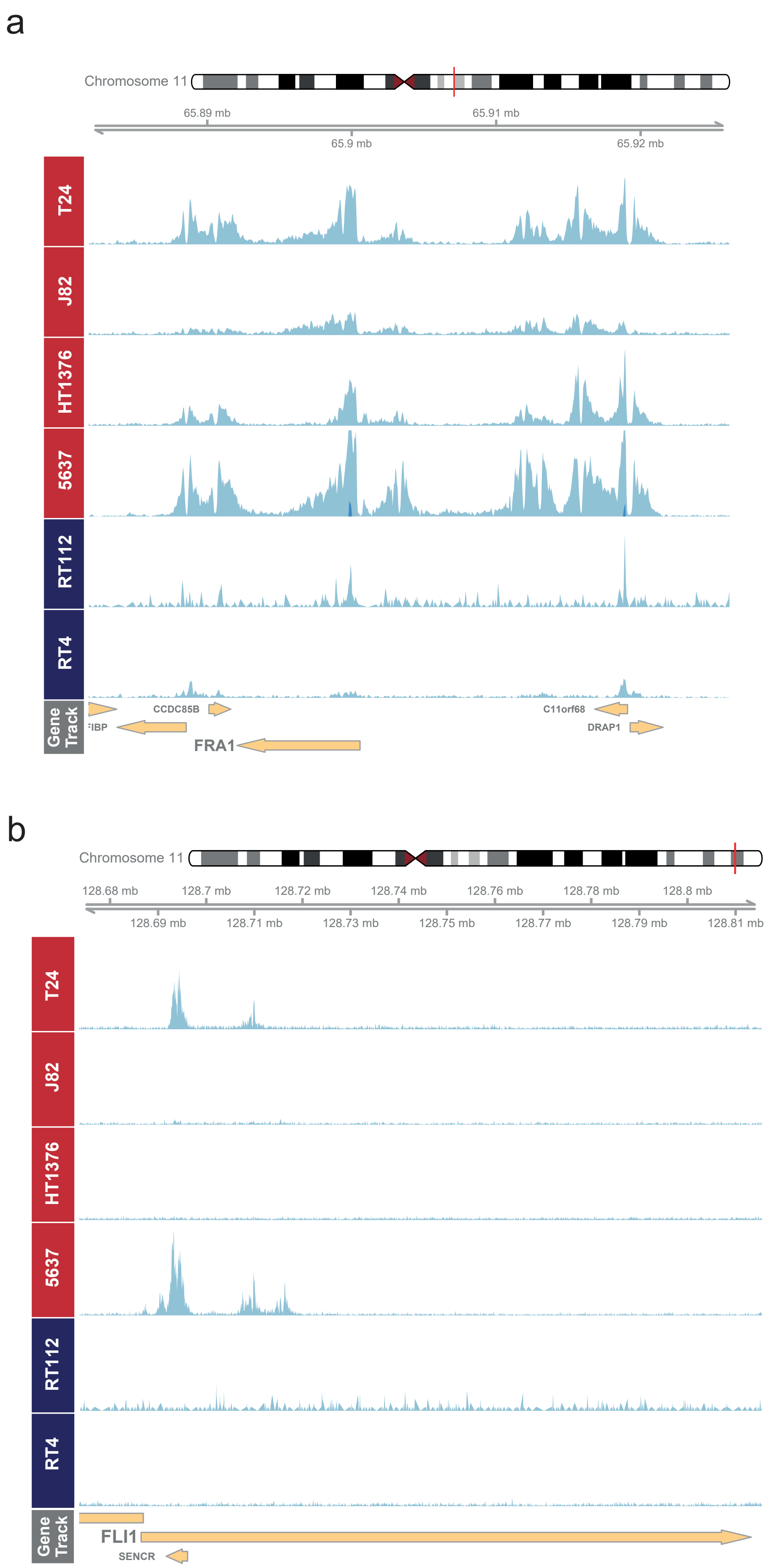

Supplementary Figure 9. H3K27ac occupancy profiles of FRA1 and FLI1. Snapshots showing H3K27ac signal in MIBC and NMIBC cell lines at *FRA1* (a) and *FLI1* (b) loci. Scales of the snapshots are adjusted to 50 and 35, respectively.

a

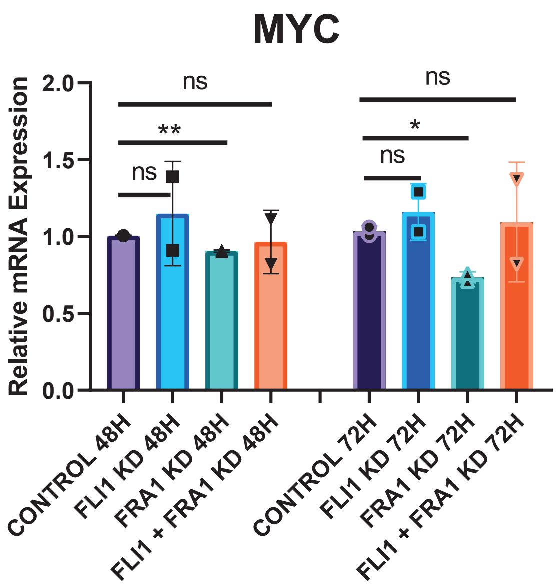

b

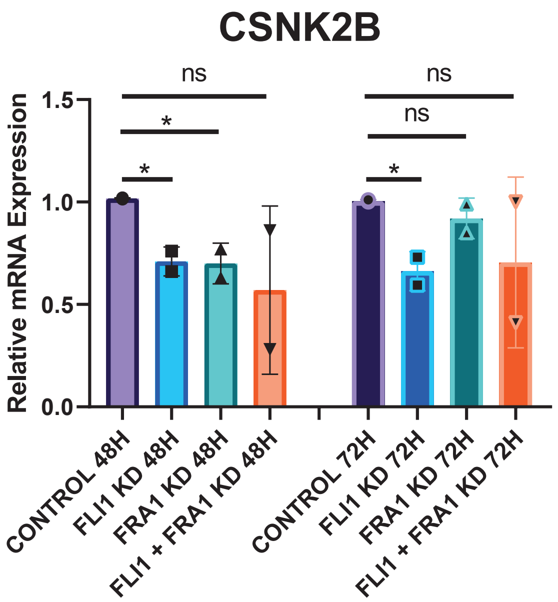

c

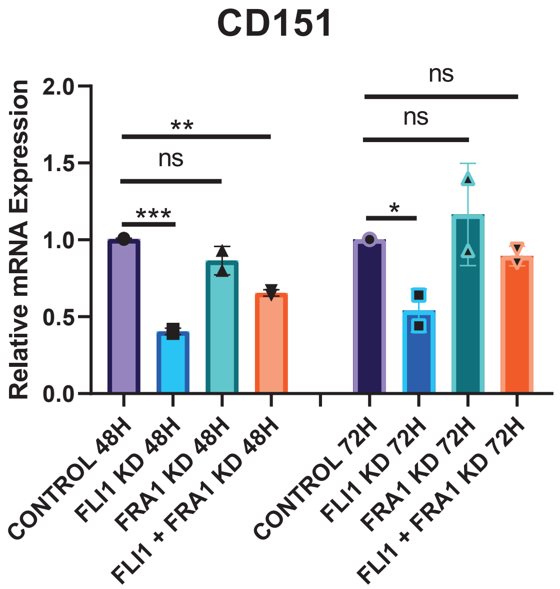

Supplementary Figure 10. Expression level of EMT-related genes after FRA1 and FLI1 knockdown. (a-c) Barplots display the relative mRNA levels of MYC (a), CSNK2B (b) and CD151 (c). Error bars show the standard deviation of two biological replicates. Each biological replicate is analyzed in three technical replicates.* p value < 0.05, ** p value < 0.01, *** p value < 0.001, ns = not significant.

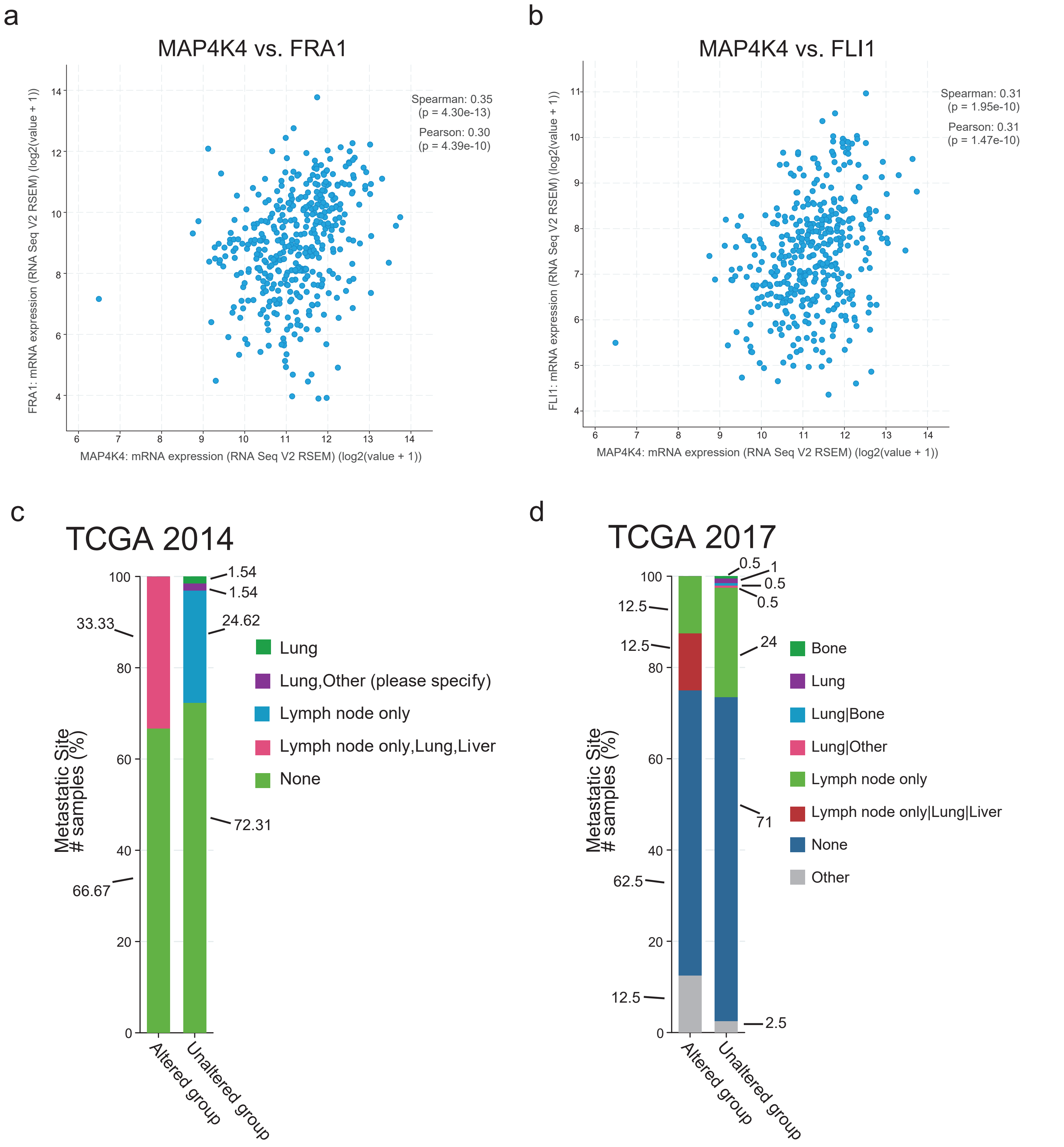

Supplementary Figure11. Association of MAP4K4 expression with metastatic potential of MIBC. (a-b) Scatter plots display the correlation between the expression of FRA1 (a) and FLI1 (b) with MAP4K4 expression in primary tissue. (c-d) The samples with high MAP4K4 expression is determined in cbioportal database. Barplots show the distribution of metastatic sites in the patients with high expression and not high MAP4K4 expression in two different cohorts,TCGA 2014 (c) (p value = 1.586e-4,q value = 5.868e-3) and TCGA 2017 (d) (p value = 1.878e-4, q value = 4.508e-3).

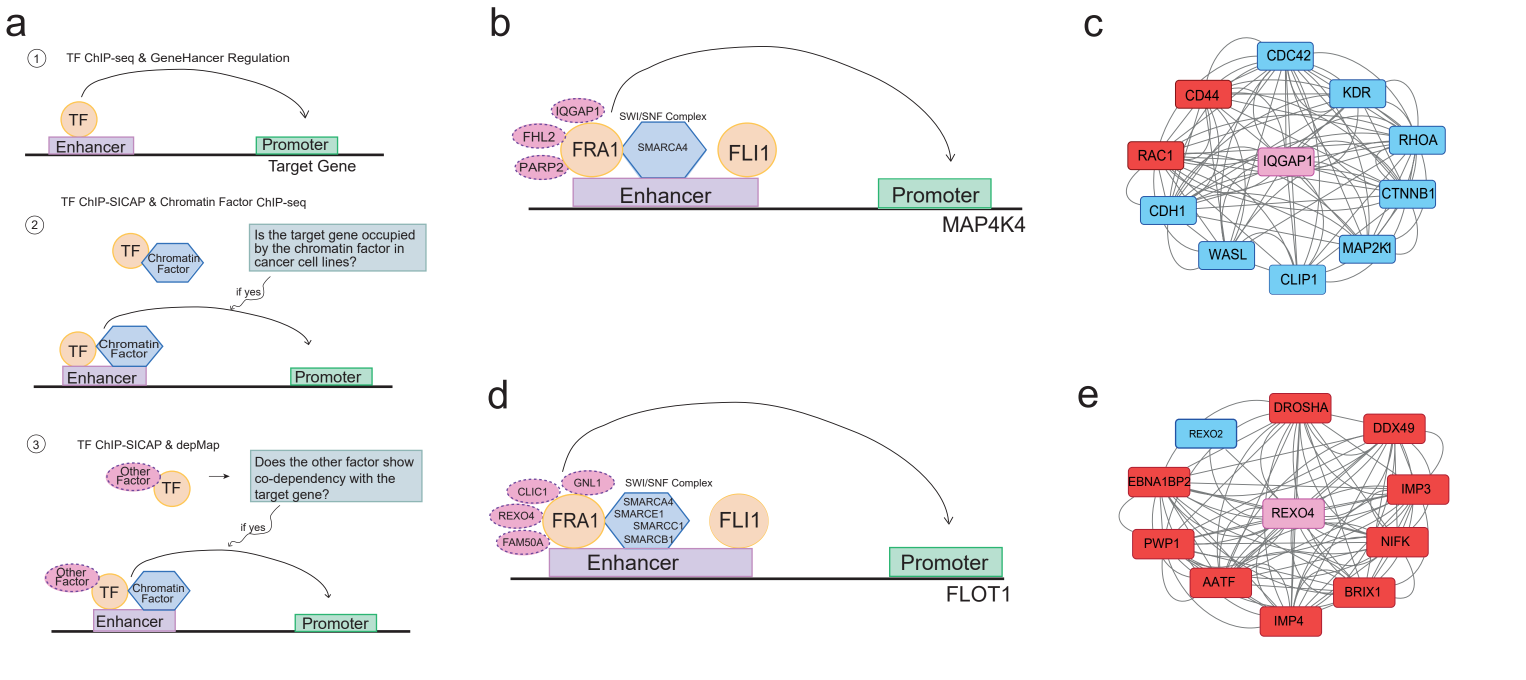

Supplementary Figure 12. Regulatory hubs implicated in regulation of EMT-related genes.
(a) Schematic showing the workflow for the constitution of the regulatory hubs. (b, d) Regulatory hubs for the regulation of MAP4K4 (b) and FLOT1 (d). (c, e) String protein-protein interaction networks for IQGAP1 (c) and REXO4 (e). Proteins which can be also identified in FRA1 chromatin-bound interactome (Supplementary Table 5) are shown in red.

a

Complex Name	Protein Name	Cell Line	Cancer Type	GEO ID	Available Data Type
SWI/SNF Complex	SMARCA4	501Mel	Melanoma	GSM1517753	wig file
SWI/SNF Complex	SMARCA4	TTC-549	Liver rhabdoid tumor derived cell line	GSM1835892	wig file
SWI/SNF Complex	SMARCE1	MCF7	Breast adenocarcinoma	GSM2827281	wig and peak file
SWI/SNF Complex	SMARCE1	MCF7	Breast adenocarcinoma	GSM2827282	wig and peak file
SWI/SNF Complex	SMARCC1	TTC-549	Liver rhabdoid tumor derived cell line	GSM1835891	wig file
SWI/SNF Complex	SMARCC1	BIN 67	Small cell carcinoma of the hypercalcemic type derived	GSM3307888	bigwig file
SWI/SNF Complex	SMARCB1	HeLa-S3	Epithelial adenocarcinoma of uterus	GSM935635	bigwig

b

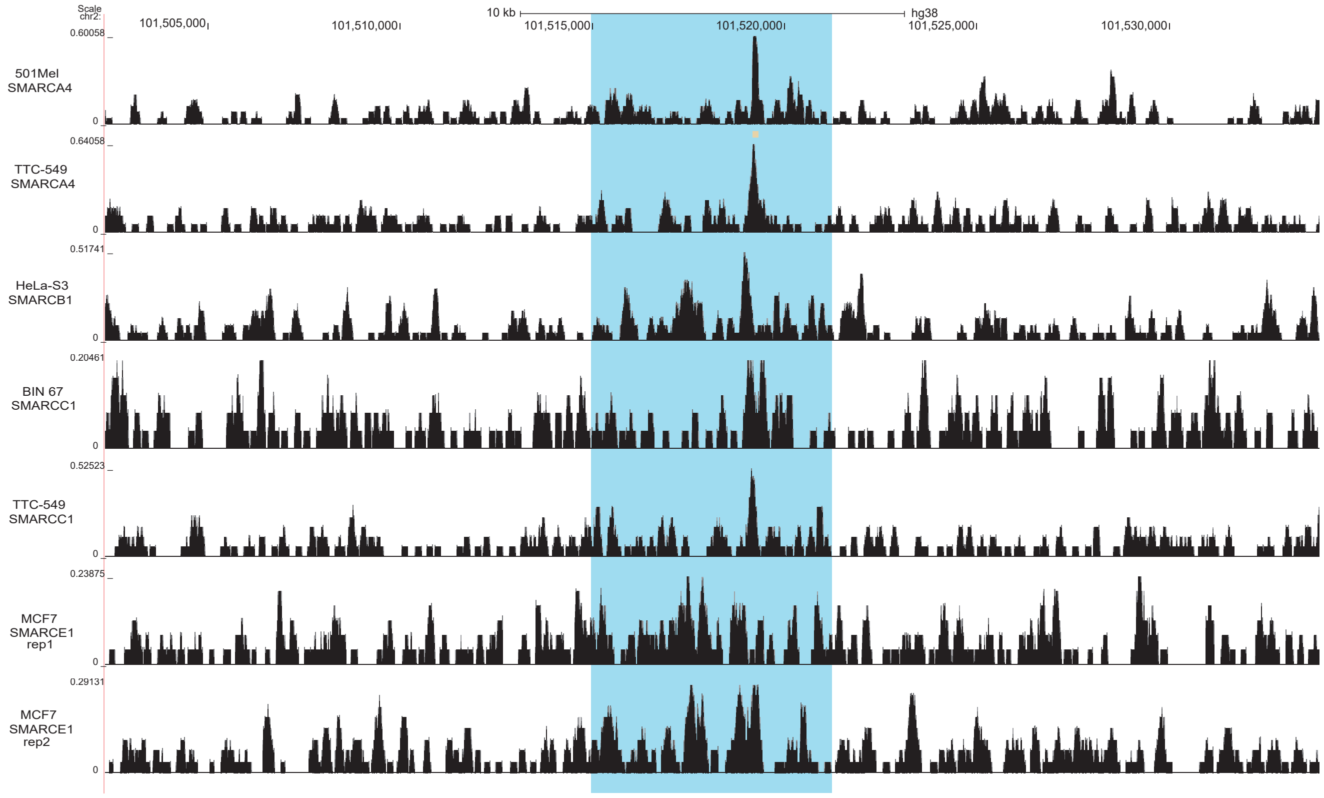

c

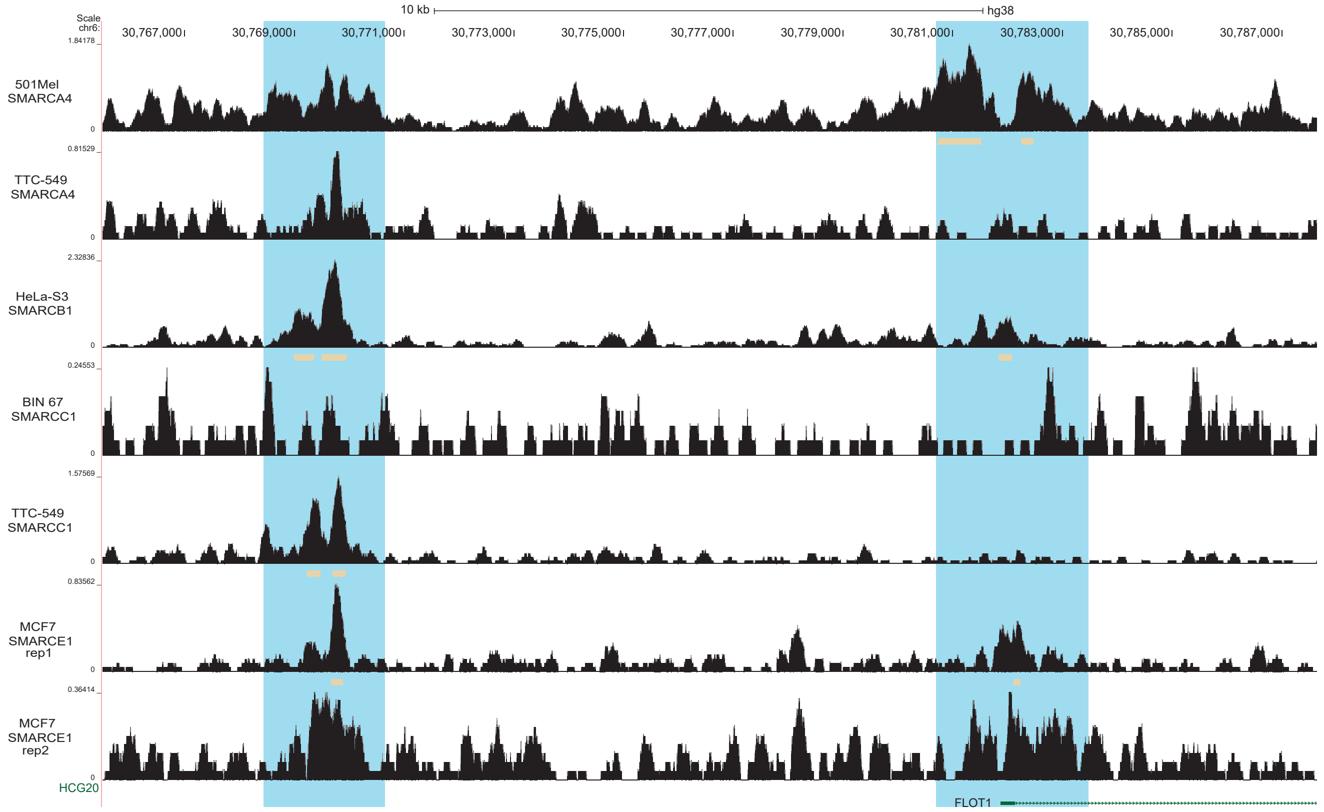

Supplementary Figure 13. ChIP-seq signal of SWI/SNF complex components at MIBC enhancers regulating MAP4K4 and FLOT1. (a) Summary of the used datasets. (b-c) Snapshot visualizations from UCSC Genome Browser, showing the localization of the SWI/SNF complex components according to data from (a) at enhancers regulating MAP4K4 (b) and FLOT1 (c). Orange rectangles below the signal track show the peak region for the respective signal. Blue highlights depict the respective enhancer regions. For FLOT1 gene, two enhancer regions involved in the regulation of this gene are shown.

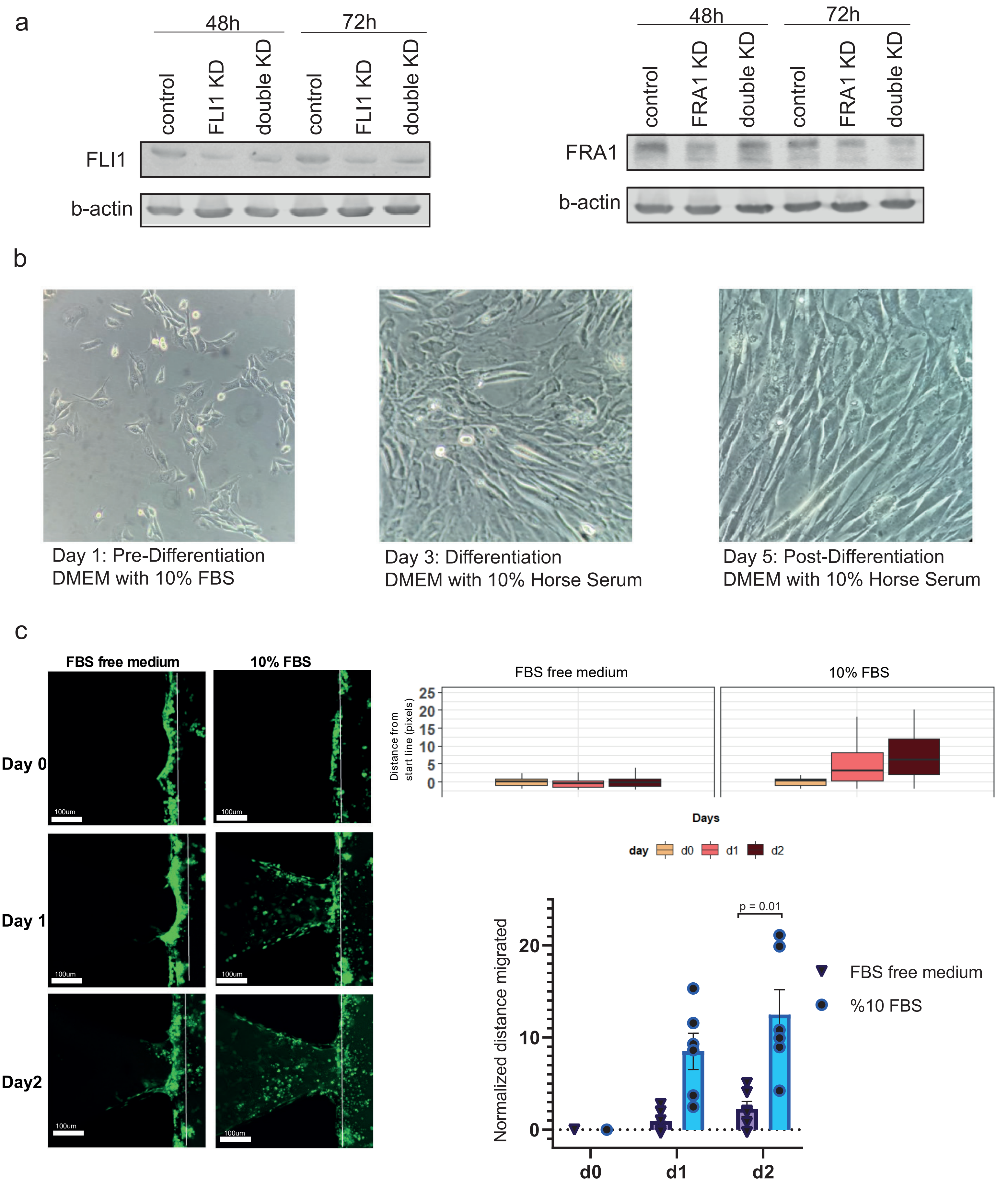

Supplementary Figure 14. Knockdown efficiency of FLI1 and FRA1 in T24 cells used in IC-CHIP and differentiation of C2C12 cells and IC-CHIP assay in the presence and absence of FBS. (a)Western blot images show the FLI1 and FRA1 levels after FLI1 and FRA1 knock-down. The images show the differentiation of C2C12 myoblast cells to muscle for 4 days in the presence of 10% horse serum. (b) Comparison of the invasive capacity of T24 cells in the presence of 0% FBS and 10% FBS. (c) Left panels show the representative Z-stack images for different conditions. Scale bar: 100µm. Boxplots display the distribution of the distance from the start line (right upper panel). Bar plots show the distance values from day 0 to day 2. In the graphs, the data is normalized to day 0 (right lower panel).

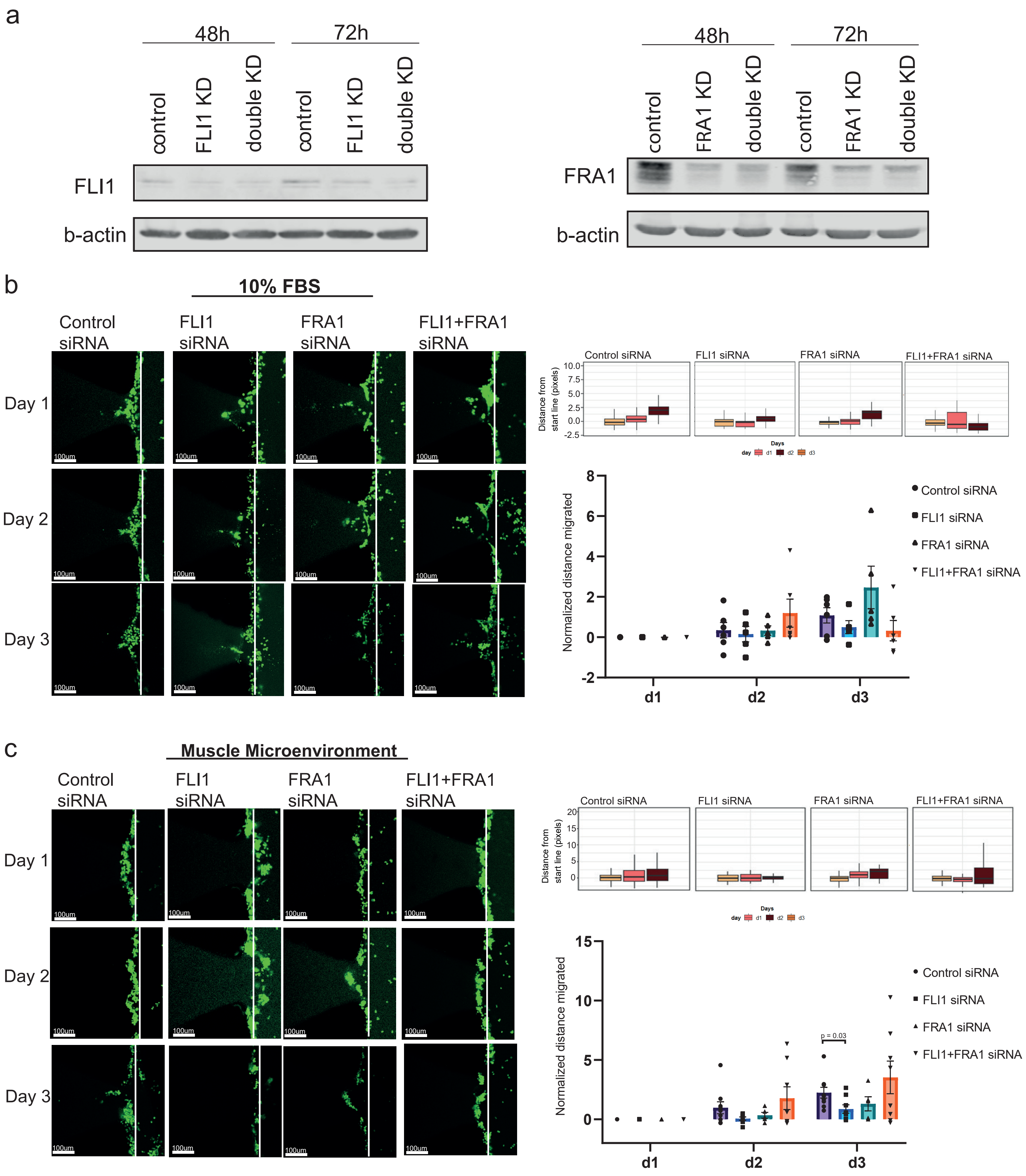

Supplementary Figure 15. Knockdown efficiency of FLI1 and FRA1 in 5637 cells and comparison of the invasive capacity of 5637 cells into cell-free matrigel in the presence of FBS and muscle microenvironment.(a) Western blot images show the FLI1 and FRA1 levels after FLI1 and FRA1 knock-down. (b-c) Comparison of the invasive capacity of 5637 cells into cell-free matrigel in the presence of FBS (b) and into the muscle microenvironment (c). Left panels show the representative Z-stack projection images for different conditions. Scale bar: 100µm. Boxplots display the distribution of the distance from the start line (right upper panel). Bar plots show the mean distance values with the error bars (n=2-6) from day 1 to day 3. The data is normalized to day 1 (right lower panel). Student's t-test (two-tailed) was used for the statistics.

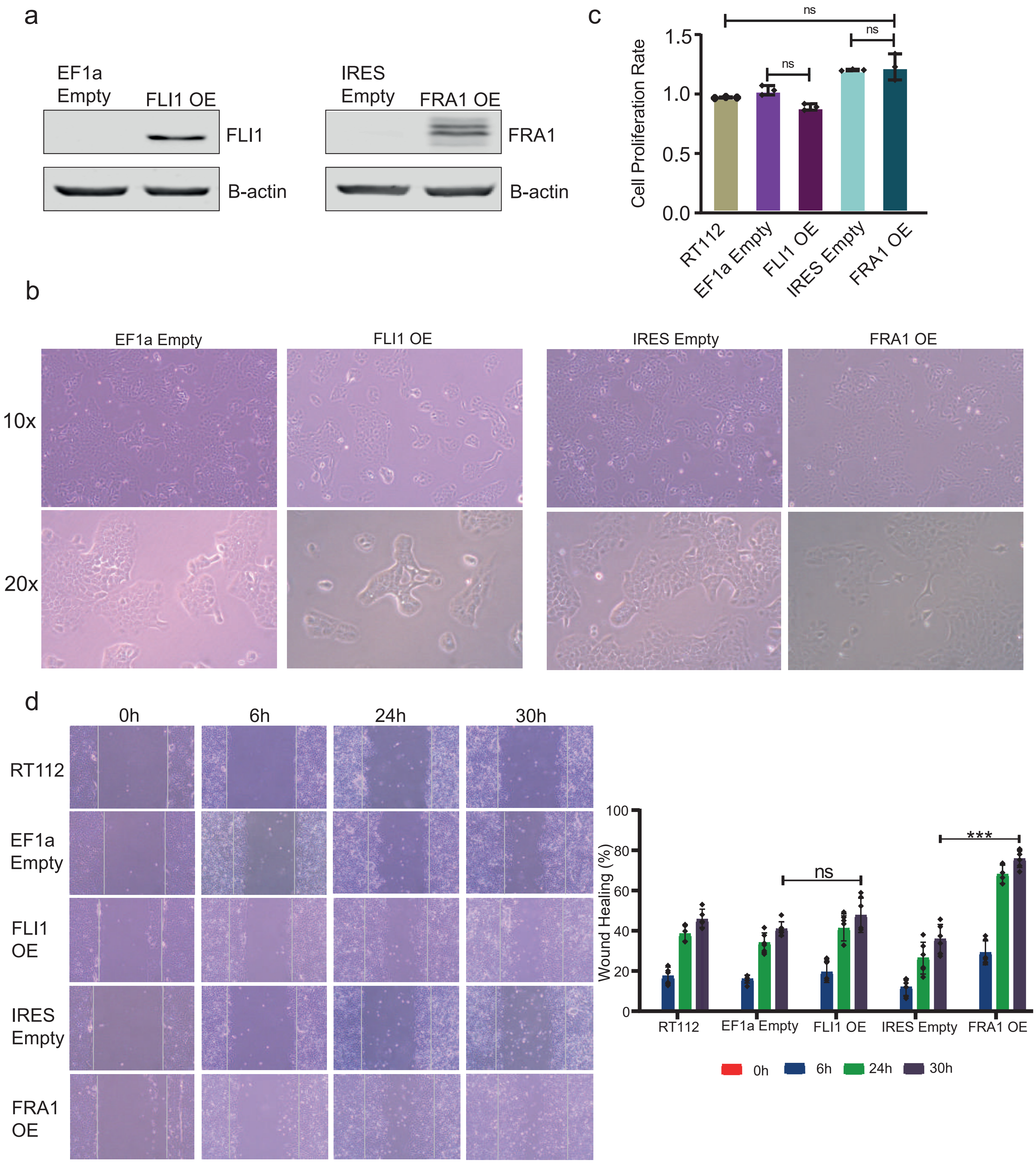

Supplementary Figure 16. Overexpression of FRA1 and FLI1 transcription factors in RT112 cell line. (a) Western blot images showing FLI1 and FRA1 protein levels for control vector and FRA1 or FLI1 overexpressed RT112 cell line. (b) Microscope images show the morphological changes between control and FRA1 or FLI1 overexpressed cells, images were taken with 10x and 20x magnification. (c) MTT cell proliferation assay results, error bars show the standard deviation of three technical replicates. (d) Wound healing scratch assay results. Left panel shows cell images taken for different time points. Images were taken with 10x magnification. The graph in right panel shows the quantification of wound healing assay results, error bars show the standard deviation of at least 5 independent measurements. *** p value < 0.0005

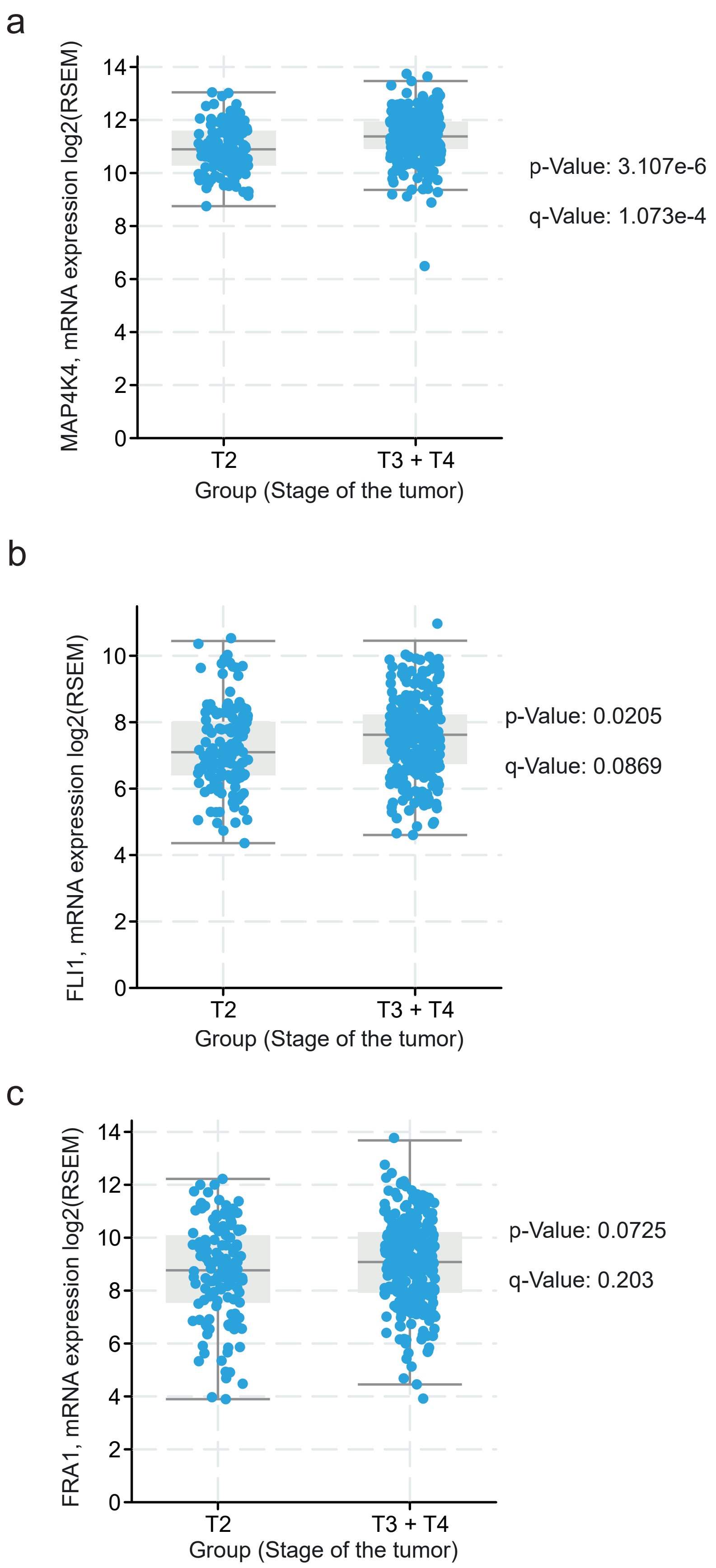

Supplementary Figure 17. Expression of key MIBC genes in T2 vs T3-T4 stage MIBC patients. (a-c) Boxplots show the expression of MAP4K4 (a), FLI1 (b) and FRA1 (c) in T2 stage and T3-T4 stage MIBC patients.

This supplementary figure contains the original Western Blot images displayed in Figure 4c-d, Figure S14a, Figure S15a and Figure S16a.

Figure 4c-d

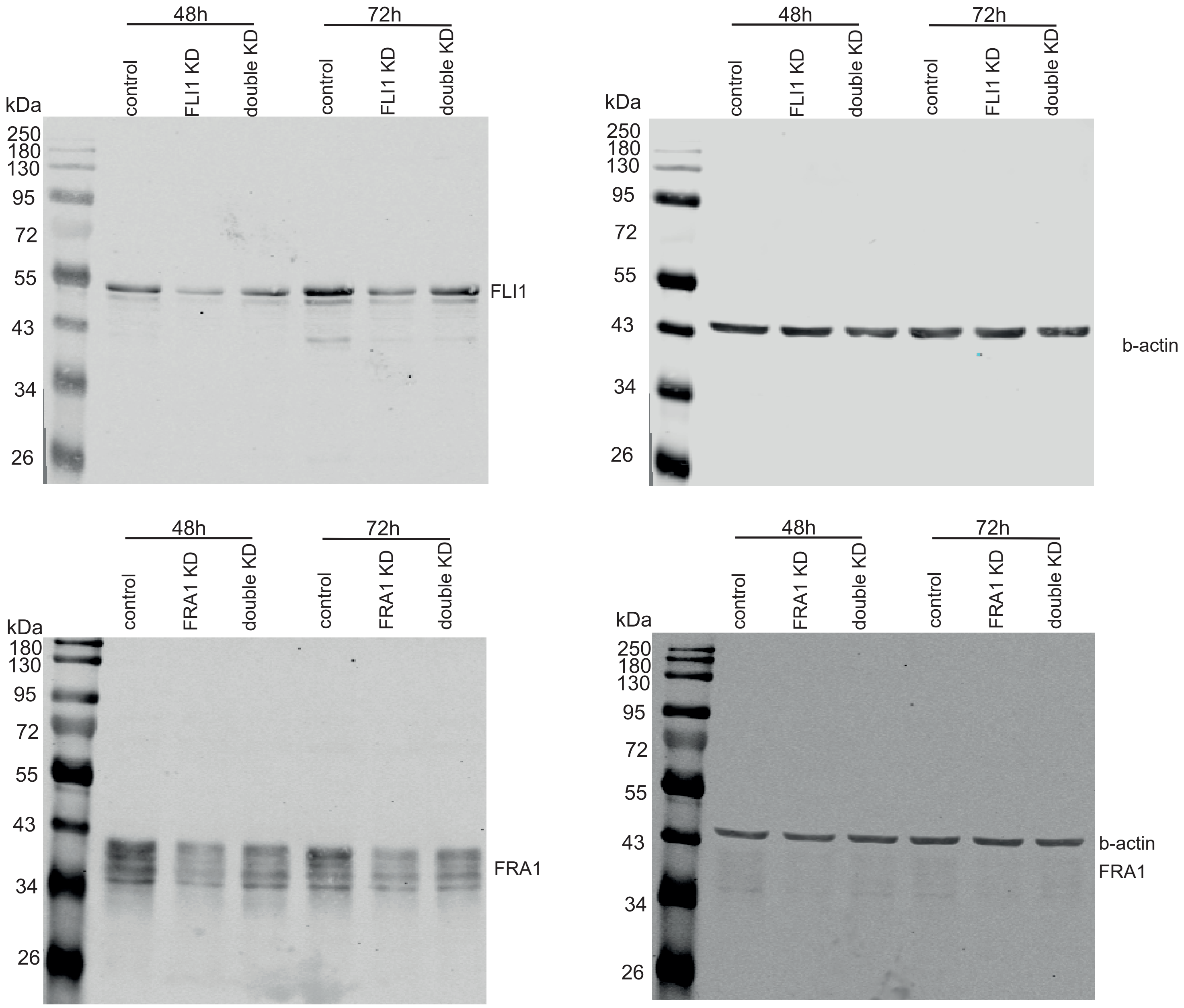

Figure S14a

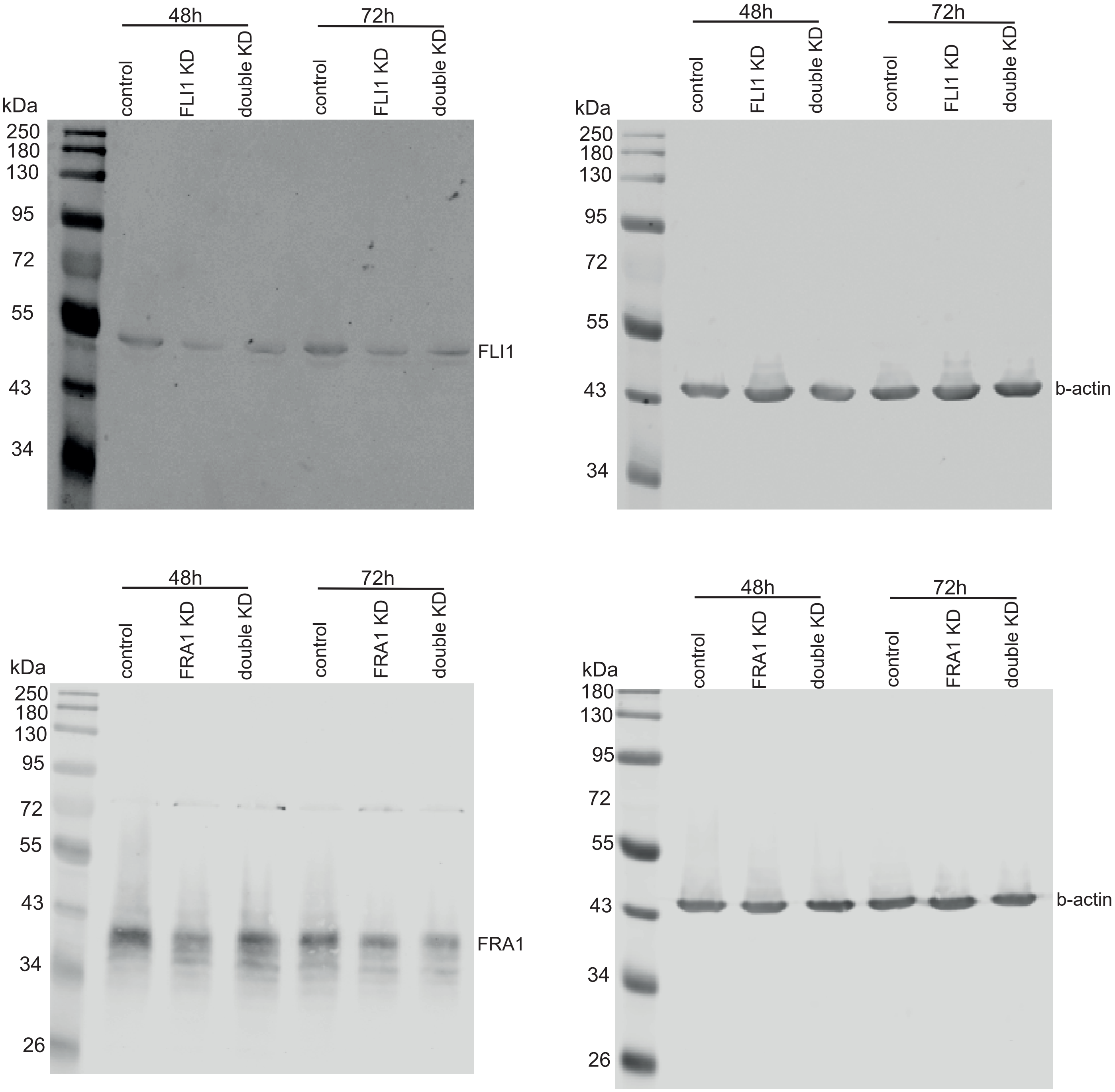
48h
72h
control
FLI1 KD
double KD
kDa
250
180
130
95
72
55
43
34
FLI1
b-actin
FRA1 KD
FRA1
26

Figure S15a

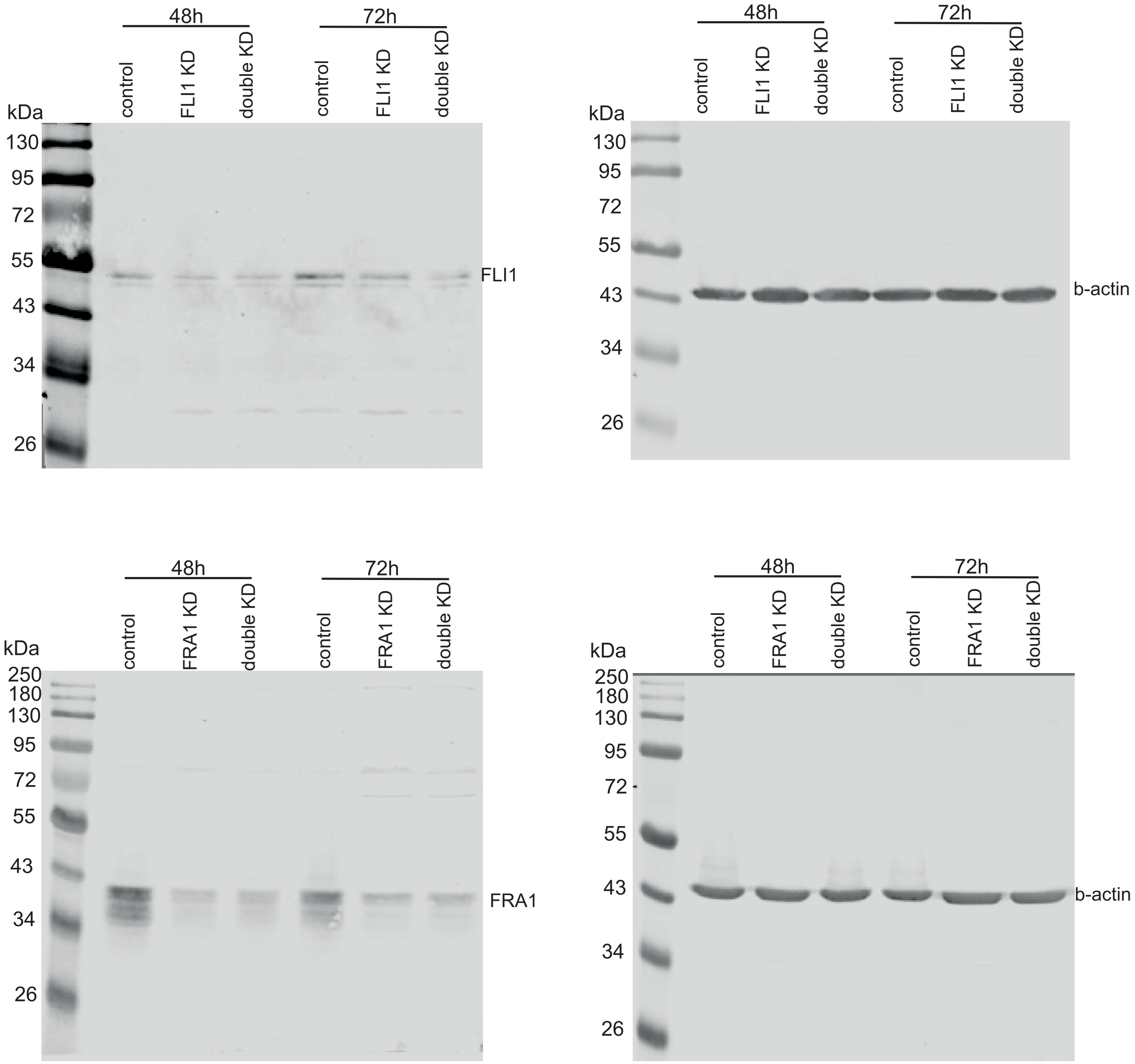
48h
72h
control
FLI1 KD
double KD
control
FLI1 KD
double KD
kDa
130
95
72
55
43
34
26
FLI1
48h
72h
control
FLI1 KD
double KD
control
FLI1 KD
double KD
kDa
130
95
72
55
43
34
26
b-actin
48h
72h
control
FRA1 KD
double KD
control
FRA1 KD
double KD
kDa
250
180
130
95
72
55
43
34
26
FRA1
48h
72h
control
FRA1 KD
double KD
control
FRA1 KD
double KD
kDa
250
180
130
95
72
55
43
34
26
b-actin

Figure S16a

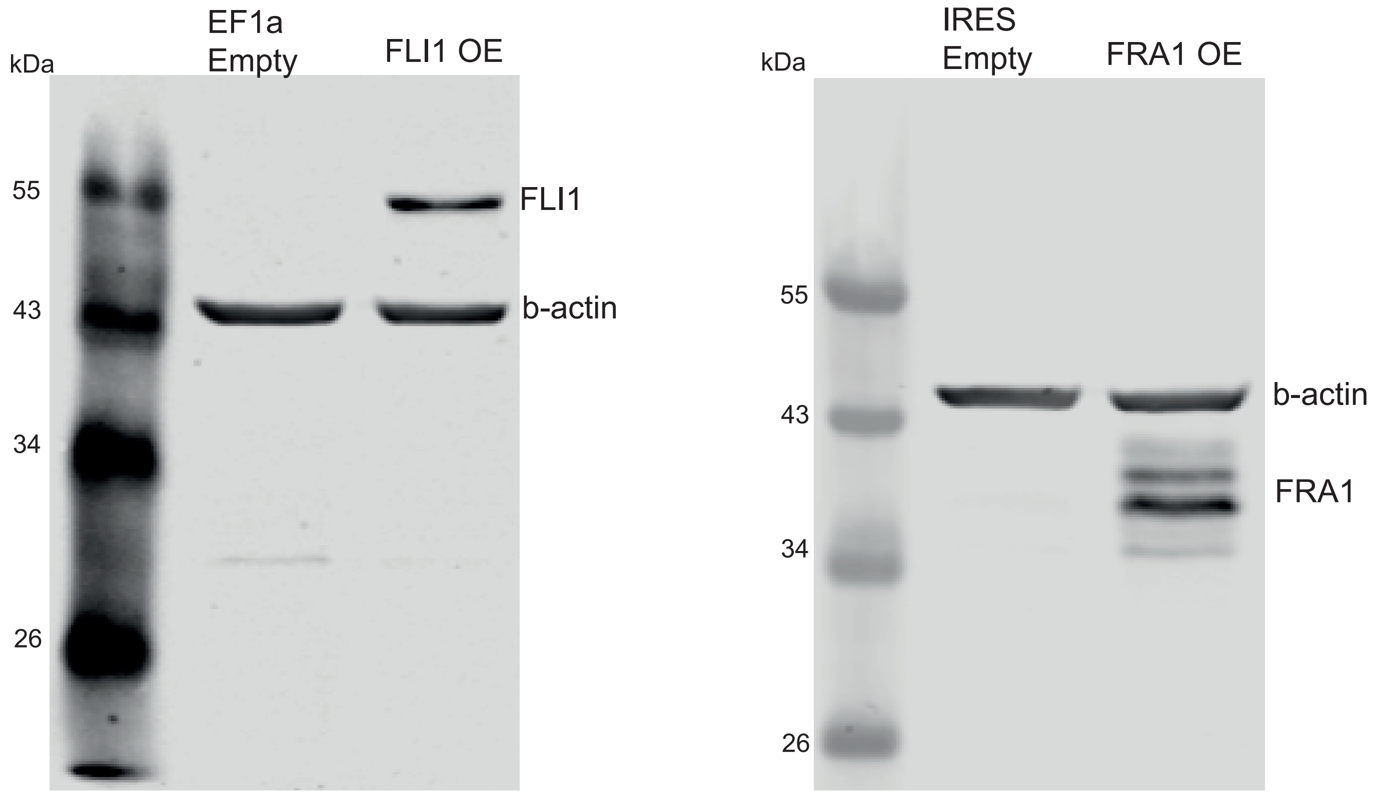

Supplementary Figure 18. Original Western Blot images displayed in Figure 4c-d, Figure S14a, Figure S15a and Figure S16a.
