# Supplementary material for: FLI1 and FRA1 transcription factors drive the transcriptional regulatory networks characterizing muscle invasive bladder cancer
Source: Commun Biol. 2023 Feb 20;6:199. doi: 10.1038/s42003-023-04561-3 (PMC9941102; doi:10.1038/s42003-023-04561-3)
Supplement: Supplementary file 2 — Description of Additional Supplementary Files [file 42003_2023_4561_MOESM2_ESM.pdf]

Description of Additional Supplementary Files

File name: Supplementary Data 1
Description: MIBC enhancers and their target genes.

File name: Supplementary Data 2
Description: NMIBC enhancers and their target genes.

File name: Supplementary Data 3
Description: Complete results of ClusterProfiler GO Term analysis for the target genes of MIBC enhancers.

File name: Supplementary Data 4
Description: List of transcription factors identified in target genes of NMIBC enhancers.

File name: Supplementary Data 5
Description: List of proteins identified to be interacting with FRA1 in T24 cell line according to ChIP-SICAP results.

File name: Supplementary Data 6
Description: List of proteins identified to be interacting with FLI1 in T24 cell line according to ChIP-SICAP results.

File name: Supplementary Data 7
Description: Overlapped Protein Interactors of FLI1 & FRA1 Transcription Factors.

File name: Supplementary Data 8
Description: Primers used in RT-qPCR experiments.

File name: Supplementary Data 9
Description: Source data of main figures' graphs.
