# Supplementary material for: FLI1 and FRA1 transcription factors drive the transcriptional regulatory networks characterizing muscle invasive bladder cancer
Source: Commun Biol. 2023 Feb 20;6:199. doi: 10.1038/s42003-023-04561-3 (PMC9941102; doi:10.1038/s42003-023-04561-3)
Supplement: Supplementary file 12 — Reporting Summary [file 42003_2023_4561_MOESM12_ESM.pdf]

nature portfolio

Corresponding author(s): Dr. Serap ERKEK-OZHAN

Last updated by author(s): Aug 10, 2022

Reporting Summary

Nature Portfolio wishes to improve the reproducibility of the work that we publish. This form provides structure for consistency and transparency in reporting. For further information on Nature Portfolio policies, see our Editorial Policies and the Editorial Policy Checklist.

Statistics

For all statistical analyses, confirm that the following items are present in the figure legend, table legend, main text, or Methods section.

n/a	Confirmed	
☐	☒	The exact sample size (*n*) for each experimental group/condition, given as a discrete number and unit of measurement
☐	☒	A statement on whether measurements were taken from distinct samples or whether the same sample was measured repeatedly
☐	☒	The statistical test(s) used AND whether they are one- or two-sided<br>*Only common tests should be described solely by name; describe more complex techniques in the Methods section.*
☐	☒	A description of all covariates tested
☒	☐	A description of any assumptions or corrections, such as tests of normality and adjustment for multiple comparisons
☐	☒	A full description of the statistical parameters including central tendency (e.g. means) or other basic estimates (e.g. regression coefficient) AND variation (e.g. standard deviation) or associated estimates of uncertainty (e.g. confidence intervals)
☐	☒	For null hypothesis testing, the test statistic (e.g. *F*, *t*, *r*) with confidence intervals, effect sizes, degrees of freedom and *P* value noted<br>*Give P values as exact values whenever suitable.*
☒	☐	For Bayesian analysis, information on the choice of priors and Markov chain Monte Carlo settings
☒	☐	For hierarchical and complex designs, identification of the appropriate level for tests and full reporting of outcomes
☒	☐	Estimates of effect sizes (e.g. Cohen's *d*, Pearson's *r*), indicating how they were calculated

Our web collection on statistics for biologists contains articles on many of the points above.

Software and code

Policy information about availability of computer code

Data collection

Details of ChIP-Seq data are provided in ChIP-Seq Data Deposition section. The details for the external data sets used in this manuscript are as follows:
cBioPortal (https://www.cbioportal.org/) was used to work with TCGA 2014 and TCGA 2017 bladder cancer samples.
Publicly available GSE32894 microarray data in GEO was used in additional analysis.
CCLE Database (https://sites.broadinstitute.org/ccle/datasets) was used to collect bladder cancer cell lines' expression data.

Data analysis

The software and the packages listed below were used in data analysis of this manuscript.
R version 3.6.0. and 4.0.5.
R Studio version 1.4.1106 and 1.1.442
Burrows Wheeler Aligner version 0.7.17-r1188
HOMER version 4.10.3.
MACS2 version 2.1.2
deepTools version 3.5.0
DiffBind Bioconductor Package
GViz Bioconductor Package
Mclust CRAN Package
biomaRt Bioconductor Package
CePa CRAN Package
tibble CRAN Package
Limma Bioconductor Package
ggplot2 CRAN Package
ggsignif CRAN Package
ggrepel CRAN Package
ggthemes CRAN Package

GenomicRanges Bioconductor Package
org.Hs.eg.db. Bioconductor Package (version 3.8.2)
clusterProfiler Bioconductor Package
rtracklayer Bioconductor Package
ggVennDiagram CRAN Package
dplyr CRAN Package
Gephi version 0.9.2
MaxQuant version 1.6.2.6
GRAPHPAD Prism version 8.3.0
Image Studio Lite version 5.2.5
Python version
Image J version 1.53f (Java 1.8.0_112 (64 bit))
Cytoscape version 3.8.2
cBioPortal (https://www.cbioportal.org/)
Cistrome Data Browser (http://www.cistrome.org/db/#/)
depMap (https://depmap.org/portal/)
String Database (https://string-db.org/)
UCSC Table Browser (https://genome.ucsc.edu/cgi-bin/hgTables)

For manuscripts utilizing custom algorithms or software that are central to the research but not yet described in published literature, software must be made available to editors and reviewers. We strongly encourage code deposition in a community repository (e.g. GitHub). See the Nature Portfolio guidelines for submitting code & software for further information.

Data

Policy information about availability of data

All manuscripts must include a data availability statement. This statement should provide the following information, where applicable:

- Accession codes, unique identifiers, or web links for publicly available datasets
- A description of any restrictions on data availability
- For clinical datasets or third party data, please ensure that the statement adheres to our policy

ChIP-seq data has been deposited in NCBI Gene Expression Omnibus (GEO) database with accession number GSE213533. ChIP-SICAP mass spectrometry proteomics data have been deposited to the ProteomeXchange Consortium (http://proteomecentral.proteomexchange.org) via the PRIDE (Perez-Riverol et al., 2019) partner repository with the dataset identifier PXD028665.

Field-specific reporting

Please select the one below that is the best fit for your research. If you are not sure, read the appropriate sections before making your selection.

☒ Life sciences ☐ Behavioural & social sciences ☐ Ecological, evolutionary & environmental sciences

For a reference copy of the document with all sections, see nature.com/documents/nr-reporting-summary-flat.pdf

Life sciences study design

All studies must disclose on these points even when the disclosure is negative.

Sample size	Six bladder cancer cell lines were used. Two of them are non muscle invasive bladder cancer cell lines and four of them are muscle invasive bladder cancer cell lines.
Data exclusions	There is no exclusion needed.
Replication	H3K27ac ChIP-Seq :<br>4 cell lines in MIBC and 2 cell lines in NMIBC were used as biological replicates of each group. Non-muscle invasive bladder cancer cell lines (NMIBC) consist of the cell lines, RT4 and RT112, and muscle invasive cell lines consist of the cell lines, 5637, HT1376, J82 and T24.<br>Transcription Factor ChIP-Seq:<br>FRA1 and FLI1 ChIP-Seq experiments were performed in T24 cell line (2 biological replicate for each of them).<br>ChIP-SICAP:<br>FRA1 and FLI1 ChIP-SICAP experiments were performed in T24 cell line (2 biological replicates for each factor).<br>FLI1 and FRA1 unique and co-knockdown experiments were performed in T24 and 5637 cell line (at least 2 biological replicates for each condition)<br>IC-CHIP experiments were performed in T24 and 5637 cell line (at least 2 biological replicates for each condition)
Randomization	*Describe how samples/organisms/participants were allocated into experimental groups. If allocation was not random, describe how covariates were controlled OR if this is not relevant to your study, explain why.*
Blinding	Since we worked with commercial cell lines, blindness was not needed.

Behavioural & social sciences study design

All studies must disclose on these points even when the disclosure is negative.

Study description	*Briefly describe the study type including whether data are quantitative, qualitative, or mixed-methods (e.g. qualitative cross-sectional, quantitative experimental, mixed-methods case study).*
Research sample	*State the research sample (e.g. Harvard university undergraduates, villagers in rural India) and provide relevant demographic information (e.g. age, sex) and indicate whether the sample is representative. Provide a rationale for the study sample chosen. For studies involving existing datasets, please describe the dataset and source.*
Sampling strategy	*Describe the sampling procedure (e.g. random, snowball, stratified, convenience). Describe the statistical methods that were used to predetermine sample size OR if no sample-size calculation was performed, describe how sample sizes were chosen and provide a rationale for why these sample sizes are sufficient. For qualitative data, please indicate whether data saturation was considered, and what criteria were used to decide that no further sampling was needed.*
Data collection	*Provide details about the data collection procedure, including the instruments or devices used to record the data (e.g. pen and paper, computer, eye tracker, video or audio equipment) whether anyone was present besides the participant(s) and the researcher, and whether the researcher was blind to experimental condition and/or the study hypothesis during data collection.*
Timing	*Indicate the start and stop dates of data collection. If there is a gap between collection periods, state the dates for each sample cohort.*
Data exclusions	*If no data were excluded from the analyses, state so OR if data were excluded, provide the exact number of exclusions and the rationale behind them, indicating whether exclusion criteria were pre-established.*
Non-participation	*State how many participants dropped out/declined participation and the reason(s) given OR provide response rate OR state that no participants dropped out/declined participation.*
Randomization	*If participants were not allocated into experimental groups, state so OR describe how participants were allocated to groups, and if allocation was not random, describe how covariates were controlled.*

Ecological, evolutionary & environmental sciences study design

All studies must disclose on these points even when the disclosure is negative.

Study description	*Briefly describe the study. For quantitative data include treatment factors and interactions, design structure (e.g. factorial, nested, hierarchical), nature and number of experimental units and replicates.*
Research sample	*Describe the research sample (e.g. a group of tagged Passer domesticus, all Stenocereus thurberi within Organ Pipe Cactus National Monument), and provide a rationale for the sample choice. When relevant, describe the organism taxa, source, sex, age range and any manipulations. State what population the sample is meant to represent when applicable. For studies involving existing datasets, describe the data and its source.*
Sampling strategy	*Note the sampling procedure. Describe the statistical methods that were used to predetermine sample size OR if no sample-size calculation was performed, describe how sample sizes were chosen and provide a rationale for why these sample sizes are sufficient.*
Data collection	*Describe the data collection procedure, including who recorded the data and how.*
Timing and spatial scale	*Indicate the start and stop dates of data collection, noting the frequency and periodicity of sampling and providing a rationale for these choices. If there is a gap between collection periods, state the dates for each sample cohort. Specify the spatial scale from which the data are taken*
Data exclusions	*If no data were excluded from the analyses, state so OR if data were excluded, describe the exclusions and the rationale behind them, indicating whether exclusion criteria were pre-established.*
Reproducibility	*Describe the measures taken to verify the reproducibility of experimental findings. For each experiment, note whether any attempts to repeat the experiment failed OR state that all attempts to repeat the experiment were successful.*
Randomization	*Describe how samples/organisms/participants were allocated into groups. If allocation was not random, describe how covariates were controlled. If this is not relevant to your study, explain why.*
Blinding	*Describe the extent of blinding used during data acquisition and analysis. If blinding was not possible, describe why OR explain why blinding was not relevant to your study.*

Did the study involve field work? ☐ Yes ☐ No

Field work, collection and transport

Field conditions	*Describe the study conditions for field work, providing relevant parameters (e.g. temperature, rainfall).*
Location	*State the location of the sampling or experiment, providing relevant parameters (e.g. latitude and longitude, elevation, water depth).*
Access & import/export	*Describe the efforts you have made to access habitats and to collect and import/export your samples in a responsible manner and in compliance with local, national and international laws, noting any permits that were obtained (give the name of the issuing authority, the date of issue, and any identifying information).*
Disturbance	*Describe any disturbance caused by the study and how it was minimized.*

Reporting for specific materials, systems and methods

We require information from authors about some types of materials, experimental systems and methods used in many studies. Here, indicate whether each material, system or method listed is relevant to your study. If you are not sure if a list item applies to your research, read the appropriate section before selecting a response.

Materials & experimental systems

n/a	Involved in the study	
☐	☒	Antibodies
☐	☒	Eukaryotic cell lines
☐	☐	Palaeontology and archaeology
☐	☐	Animals and other organisms
☐	☐	Human research participants
☐	☐	Clinical data
☐	☐	Dual use research of concern

Methods

n/a	Involved in the study	
☐	☒	ChIP-seq
☐	☐	Flow cytometry
☐	☐	MRI-based neuroimaging

Antibodies

Antibodies used	H3K27ac Antibody, Active Motif, Catalog Number #39133, Lot Number #01518010<br>FLI1 antibody, Abcam, Catalog Number #ab15289, Lot Number #GR3229309-7<br>FRA1 antibody, Cell Signaling Technologies, Catalog Number #5281, Lot Number #2, Clone Number #D80B4<br>Beta-actin antibody, Cell Signaling Technologies,Catalog Number #3700, Lot Number #17, clone number : 8H10D10<br>IRDye 680 Mouse, LI-COR, Catalog Number 926-68020, Lot Number #C90521-09<br>Anti-rabbit IgG (H+L) (DyLight™ 800) antibody, Cell Signaling Technologies, Catalog Number #5151, Lot Number #12<br>FLI1 antibody for rep2, Cell Signaling Technologies, Catalog Number #35980, Lot Number #1 , Clone Number #D7N5M
Validation	Rabbit H3K27ac Antibody, Applications Validated by Active Motif: ChIP: 10 µg per ChIP, ChIP-Seq: 5 µg each, ICC/IF: 1 - 5 µg/ml dilution, WB*: 0.1 - 1 µg/ml dilution, CUT&Tag: 1 µg per 50 µl reaction*<br>Rabbit FLI1 antibody, ChIP application is validated by this manuscript and as an example you can check another article in the following link: https://europepmc.org/backend/ptpmcrender.fcgi?accid=PMC7953068&blobtype=pdf. WB application is validated by manufacturer.<br>Rabbit FRA1 antibody, WB application is validated by manufacturer. ChIP application is validated with this article and also the following article https://www.nature.com/articles/s41598-021-94072-0.<br>Mouse β-Actin, WB application is validated by manufacturer.<br>FLI1 antibody for rep2, ChIP application is validated by manufacturer.

Eukaryotic cell lines

Policy information about [cell lines](#)

Cell line source(s)	5637 cell line - DSMZ<br>HT1376 cell line - DSMZ<br>J82 cell line - ATCC<br>RT4 cell line - ATCC<br>RT112 cell line - DSMZ<br>T24 - ATCC
Authentication	Since the cell lines were purchased from the appropriate companies known for cell line research and they were only used in our lab and research center, additional authentication was not applied.
Mycoplasma contamination	All used cell lines are negative for mycoplasma contamination.
Commonly misidentified lines (See ICLAC register)	There are not any misidentified cell lines in this study.

Palaeontology and Archaeology

Specimen provenance	*Provide provenance information for specimens and describe permits that were obtained for the work (including the name of the issuing authority, the date of issue, and any identifying information). Permits should encompass collection and, where applicable, export.*
Specimen deposition	*Indicate where the specimens have been deposited to permit free access by other researchers.*
Dating methods	*If new dates are provided, describe how they were obtained (e.g. collection, storage, sample pretreatment and measurement), where they were obtained (i.e. lab name), the calibration program and the protocol for quality assurance OR state that no new dates are provided.*

☐ Tick this box to confirm that the raw and calibrated dates are available in the paper or in Supplementary Information.

Ethics oversight	*Identify the organization(s) that approved or provided guidance on the study protocol, OR state that no ethical approval or guidance was required and explain why not.*

Note that full information on the approval of the study protocol must also be provided in the manuscript.

Animals and other organisms

Policy information about studies involving animals; ARRIVE guidelines recommended for reporting animal research

Laboratory animals	*For laboratory animals, report species, strain, sex and age OR state that the study did not involve laboratory animals.*
Wild animals	*Provide details on animals observed in or captured in the field; report species, sex and age where possible. Describe how animals were caught and transported and what happened to captive animals after the study (if killed, explain why and describe method; if released, say where and when) OR state that the study did not involve wild animals.*
Field-collected samples	*For laboratory work with field-collected samples, describe all relevant parameters such as housing, maintenance, temperature, photoperiod and end-of-experiment protocol OR state that the study did not involve samples collected from the field.*
Ethics oversight	*Identify the organization(s) that approved or provided guidance on the study protocol, OR state that no ethical approval or guidance was required and explain why not.*

Note that full information on the approval of the study protocol must also be provided in the manuscript.

Human research participants

Policy information about studies involving human research participants

Population characteristics	*Describe the covariate-relevant population characteristics of the human research participants (e.g. age, gender, genotypic information, past and current diagnosis and treatment categories). If you filled out the behavioural & social sciences study design questions and have nothing to add here, write "See above."*
Recruitment	*Describe how participants were recruited. Outline any potential self-selection bias or other biases that may be present and how these are likely to impact results.*
Ethics oversight	*Identify the organization(s) that approved the study protocol.*

Note that full information on the approval of the study protocol must also be provided in the manuscript.

Clinical data

Policy information about clinical studies

All manuscripts should comply with the ICMJE guidelines for publication of clinical research and a completed CONSORT checklist must be included with all submissions.

Clinical trial registration	*Provide the trial registration number from ClinicalTrials.gov or an equivalent agency.*
Study protocol	*Note where the full trial protocol can be accessed OR if not available, explain why.*
Data collection	*Describe the settings and locales of data collection, noting the time periods of recruitment and data collection.*
Outcomes	*Describe how you pre-defined primary and secondary outcome measures and how you assessed these measures.*

Dual use research of concern

Policy information about dual use research of concern

Hazards

Could the accidental, deliberate or reckless misuse of agents or technologies generated in the work, or the application of information presented in the manuscript, pose a threat to:

No	Yes	
☒	☐	Public health
☒	☐	National security
☒	☐	Crops and/or livestock
☒	☐	Ecosystems
☒	☐	Any other significant area

Experiments of concern

Does the work involve any of these experiments of concern:

No	Yes	
☒	☐	Demonstrate how to render a vaccine ineffective
☒	☐	Confer resistance to therapeutically useful antibiotics or antiviral agents
☒	☐	Enhance the virulence of a pathogen or render a nonpathogen virulent
☒	☐	Increase transmissibility of a pathogen
☒	☐	Alter the host range of a pathogen
☒	☐	Enable evasion of diagnostic/detection modalities
☒	☐	Enable the weaponization of a biological agent or toxin
☒	☐	Any other potentially harmful combination of experiments and agents

ChIP-seq

Data deposition

☒ Confirm that both raw and final processed data have been deposited in a public database such as GEO.

☐ Confirm that you have deposited or provided access to graph files (e.g. BED files) for the called peaks.

Data access links
May remain private before publication.

ChIP-seq data has been deposited in NCBI Gene Expression Omnibus (GEO) database with accession number GSE213533.
To review GEO accession GSE213533:
https://www.ncbi.nlm.nih.gov/geo/query/acc.cgi?acc=GSE213533
Enter token yxixgsugphabrqx into the box

ChIP-SICAP mass spectrometry proteomics data have been deposited to the ProteomeXchange Consortium (http://proteomecentral.proteomexchange.org) via the PRIDE (Perez-Riverol et al., 2019) partner repository with the dataset identifier PXD028665.

Reviewer account details:

Username: reviewer_pxd028665@ebi.ac.uk Password: jBqMAkdb

Files in database submission

Files uploaded to GEO Database:
T24_cell_line_H3K27ac_ChIP.txt.gz
T24_cell_line_H3K27ac_input.txt.gz
HT1376_cell_line_H3K27ac_ChIP.txt.gz
HT1376_cell_line_H3K27ac_input.txt.gz
5637_cell_line_H3K27ac_ChIP.txt.gz
5637_cell_line_H3K27ac_input.txt.gz
J82_cell_line_H3K27ac_ChIP.txt.gz
J82_cell_line_H3K27ac_input.txt.gz
RT112_cell_line_H3K27ac_ChIP.txt.gz
RT112_cell_line_H3K27ac_input.txt.gz
RT4_cell_line_H3K27ac_ChIP.txt.gz
RT4_cell_line_H3K27ac_input.txt.gz
T24_cell_line_FRA1_ChIP_1stRep.txt.gz
T24_cell_line_FRA1_input_1stRep.txt.gz
T24_cell_line_FLI1_ChIP_1stRep.txt.gz
T24_cell_line_FLI1_input_1stRep.txt.gz
T24_cell_line_FRA1_ChIP_2ndRep_T1_1.fastq.gz
T24_cell_line_FRA1_ChIP_2ndRep_T1_2.fastq.gz
T24_cell_line_FRA1_input_2ndRep_T1_1.fastq.gz
T24_cell_line_FRA1_input_2ndRep_T1_2.fastq.gz
T24_cell_line_FLI1_ChIP_2ndRep_T1_1.fastq.gz
T24_cell_line_FLI1_ChIP_2ndRep_T1_2.fastq.gz

T24_cell_line_FLI1_input_2ndRep_T1_1.fastq.gz
T24_cell_line_FLI1_input_2ndRep_T1_2.fastq.gz
T24_cell_line_H3K27ac_ChIP_peaks.broadPeak
HT1376_cell_line_H3K27ac_ChIP_peaks.broadPeak
5637_cell_line_H3K27ac_ChIP_peaks.broadPeak
J82_cell_line_H3K27ac_ChIP_peaks.broadPeak
RT112_cell_line_H3K27ac_ChIP_peaks.broadPeak
RT4_cell_line_H3K27ac_ChIP_peaks.broadPeak
T24_cell_line_FRA1_ChIP_peaks.bed
T24_cell_line_FLI1_ChIP_peaks.bed

Files uploaded to ProteomeXchange:
BCB9931_FLI_SICAP_R1.raw
BCB9931_FLI_SICAP_R2.raw
BCB9931_FRA_SICAP_R1.raw
BCB9931_FRA_SICAP_R2.raw
BCB9931_noAB_FLI1_SICAP.raw
BCB9931_noAB_FRA1_SICAP.raw
proteinGroups_FLI.txt
proteinGroups_FRA.txt

Genome browser session
(e.g. UCSC)

Provide a link to an anonymized genome browser session for "Initial submission" and "Revised version" documents only, to enable peer review. Write "no longer applicable" for "Final submission" documents.

Methodology

Replicates

H3K27ac ChIP-Seq :
4 cell lines in MIBC and 2 cell lines in NMIBC were used as biological replicates of each group. Non-muscle invasive bladder cancer cell lines (NMIBC) consist of the cell lines, RT4 and RT112, and muscle invasive cell lines consist of the cell lines, 5637, HT1376, J82 and T24.
Transcription Factor ChIP-Seq:
FRA1 and FLI1 ChIP-Seq experiments were performed in T24 cell line (2 biological replicate for each of them).
ChIP-SICAP:
FRA1 and FLI1 ChIP-SICAP experiments were performed in T24 cell line (2 biological replicates for each factor).

Sequencing depth

Below please find the bam file statistics:
T24 cell line H3K27ac ChIP-Seq data (length of the reads 50 bp -single end) :
total number of reads: 86798090
uniquely mapped reads: 80296505

T24 cell line H3K27ac input ChIP-Seq data (length of the reads 50 bp -single end) :
total number of reads: 62133244
uniquely mapped reads: 56253608

HT1376 cell line H3K27ac ChIP-Seq data (length of the reads 50 bp -single end) :
total number of reads: 122985602
uniquely mapped reads: 110786393

HT1376 cell line H3K27ac input ChIP-Seq data (length of the reads 50 bp -single end) :
total number of reads: 65897518
uniquely mapped reads: 60544263

5637 cell line H3K27ac ChIP-Seq data (length of the reads 50 bp -single end) :
total number of reads: 92101671
uniquely mapped reads: 85629715

5637 cell line H3K27ac input ChIP-Seq data (length of the reads 50 bp -single end) :
total number of reads: 37251256
uniquely mapped reads: 31998837

J82 cell line H3K27ac ChIP-Seq data (length of the reads 50 bp -single end) :
total number of reads: 83192638
uniquely mapped reads: 76794061

J82 cell line H3K27ac input ChIP-Seq data (length of the reads 50 bp -single end) :
total number of reads: 67945706
uniquely mapped reads: 56511252

RT112 cell line H3K27ac ChIP-Seq data (length of the reads 50 bp -single end) :
total number of reads: 15052670
uniquely mapped reads: 11011741

RT112 cell line H3K27ac input ChIP-Seq data (length of the reads 50 bp -single end) :
total number of reads: 50983489

uniquely mapped reads: 41377005

RT4 cell line H3K27ac ChIP--Seq data (length of the reads 50 bp -single end) :
total number of reads: 66497034
uniquely mapped reads: 61570207

RT4 cell line H3K27ac input ChIP-Seq data (length of the reads 50 bp -single end) :
total number of reads: 60863456
uniquely mapped reads: 55982836

T24 cell line FRA1 ChIP-Seq data rep1(length of the reads 75 bp -single end) :
total number of reads: 94225202
uniquely mapped reads: 87025280

T24 cell line FRA1 input ChIP-Seq data rep1(length of the reads 75 bp -single end) :
total number of reads: 68892646
uniquely mapped reads: 64146746

T24 cell line FLI1 ChIP-Seq data rep1 (length of the reads 75 bp -single end) :
total number of reads: 159249615
uniquely mapped reads: 146604610

T24 cell line FLI1 input ChIP-Seq data rep1 (length of the reads 75 bp -single end) :
total number of reads: 63357304
uniquely mapped reads: 58882595

T24 cell line FRA1 ChIP-Seq data rep2(length of the reads 150 bp -paired end) :
total number of reads: 79481402
uniquely mapped reads: 75412998

T24 cell line FRA1 input ChIP-Seq data rep2(length of the reads 150 bp -paired end) :
total number of reads: 79871668
uniquely mapped reads: 75672268

T24 cell line FLI1 ChIP-Seq data rep2 (length of the reads 150 bp -paired end) :
total number of reads: 79043394
uniquely mapped reads: 75015472

T24 cell line FLI1 input ChIP-Seq data rep2 (length of the reads 150 bp -paired end) :
total number of reads: 80060864
uniquely mapped reads: 75889617

Antibodies

H3K27ac Antibody, Active Motif, Catalog Number #39133, Lot Number #01518010
FLI1 antibody, Abcam, Catalog Number #ab15289, Lot Number #GR3229309-7
FRA1 antibody, Cell Signaling Technologies, Catalog Number #5281, Lot Number #2, Clone Number #D80B4
FLI1 antibody for rep2, Cell Signaling Technologies, Catalog Number #35980, Lot Number #1 , Clone Number #D7N5M

Peak calling parameters

H3K27ac peaks are called with MACS2 using callpeak command with -broad option and default parameters.
FRA1 and FLI1 transcription factor peak finding was performed using HOMER algorithm, by using two biological replicates with the code getDifferentialPeaksReplicates.pl with specifying the style as factor (-style factor), adjusting fold enrichment over input tag count to 2 (-F 2), fold enrichment over local tag count 2 (-L 2) and specifying genome to hg38.

Data quality

Quality of the ChIP-Seq data was checked using HOMER (version 4.10.3) by plotting clonal tag distribution graphs representing read distribution.
Peak qualities are:
9196 out of 32582 T24 cell line H3K27ac ChIP peaks have FDR < %5 and also have > 5-fold enrichment
10006 out of 36617 HT1376 cell line H3K27ac ChIP peaks have FDR < %5 and also have > 5-fold enrichment
12131 out of 59279 5637 cell line H3K27ac ChIP peaks have FDR < %5 and also have > 5-fold enrichment
1084 out of 9618 J82 cell line H3K27ac ChIP peaks have FDR < %5 and also have > 5-fold enrichment
9086 out of 30936 RT112 cell line H3K27ac ChIP peaks have FDR < %5 and also have > 5-fold enrichment
2958 out of 29893 RT4 cell line H3K27ac ChIP peaks have FDR < %5 and also have > 5-fold enrichment
10061 out of 10513 T24 cell line FRA1 ChIP peaks have FDR < %5 and also have > 5-fold enrichment
847 out of 2775 T24 cell line FLI1 ChIP peaks have FDR < %5 and also have > 5-fold enrichment

Software

R version 3.6.0. and 4.0.5.
R Studio version 1.4.1106
Burrows Wheeler Aligner version 0.7.17-r1188
HOMER version 4.10.3.
MACS2 version 2.1.2
deepTools version 3.5.0

DiffBind Bioconductor Package
GViz Bioconductor Package
biomaRt Bioconductor Package
tibble CRAN Package
ggplot2 CRAN Package
GenomicRanges Bioconductor Package
org.Hs.eg.db. Bioconductor Package (version 3.8.2)
clusterProfiler Bioconductor Package
rtracklayer Bioconductor Package
dplyr CRAN Package

Flow Cytometry

Plots

Confirm that:

☐ The axis labels state the marker and fluorochrome used (e.g. CD4-FITC).

☐ The axis scales are clearly visible. Include numbers along axes only for bottom left plot of group (a 'group' is an analysis of identical markers).

☐ All plots are contour plots with outliers or pseudocolor plots.

☐ A numerical value for number of cells or percentage (with statistics) is provided.

Methodology

Sample preparation	*Describe the sample preparation, detailing the biological source of the cells and any tissue processing steps used.*
Instrument	*Identify the instrument used for data collection, specifying make and model number.*
Software	*Describe the software used to collect and analyze the flow cytometry data. For custom code that has been deposited into a community repository, provide accession details.*
Cell population abundance	*Describe the abundance of the relevant cell populations within post-sort fractions, providing details on the purity of the samples and how it was determined.*
Gating strategy	*Describe the gating strategy used for all relevant experiments, specifying the preliminary FSC/SSC gates of the starting cell population, indicating where boundaries between "positive" and "negative" staining cell populations are defined.*

☐ Tick this box to confirm that a figure exemplifying the gating strategy is provided in the Supplementary Information.

Magnetic resonance imaging

Experimental design

Design type	*Indicate task or resting state; event-related or block design.*
Design specifications	*Specify the number of blocks, trials or experimental units per session and/or subject, and specify the length of each trial or block (if trials are blocked) and interval between trials.*
Behavioral performance measures	*State number and/or type of variables recorded (e.g. correct button press, response time) and what statistics were used to establish that the subjects were performing the task as expected (e.g. mean, range, and/or standard deviation across subjects).*

Acquisition

Imaging type(s)	*Specify: functional, structural, diffusion, perfusion.*
Field strength	*Specify in Tesla*
Sequence & imaging parameters	*Specify the pulse sequence type (gradient echo, spin echo, etc.), imaging type (EPI, spiral, etc.), field of view, matrix size, slice thickness, orientation and TE/TR/flip angle.*
Area of acquisition	*State whether a whole brain scan was used OR define the area of acquisition, describing how the region was determined.*

Diffusion MRI ☐ Used ☐ Not used

Preprocessing

Preprocessing software	*Provide detail on software version and revision number and on specific parameters (model/functions, brain extraction, segmentation, smoothing kernel size, etc.).*

Normalization	*If data were normalized/standardized, describe the approach(es): specify linear or non-linear and define image types used for transformation OR indicate that data were not normalized and explain rationale for lack of normalization.*
Normalization template	*Describe the template used for normalization/transformation, specifying subject space or group standardized space (e.g. original Talairach, MNI305, ICBM152) OR indicate that the data were not normalized.*
Noise and artifact removal	*Describe your procedure(s) for artifact and structured noise removal, specifying motion parameters, tissue signals and physiological signals (heart rate, respiration).*
Volume censoring	*Define your software and/or method and criteria for volume censoring, and state the extent of such censoring.*

Statistical modeling & inference

Model type and settings	*Specify type (mass univariate, multivariate, RSA, predictive, etc.) and describe essential details of the model at the first and second levels (e.g. fixed, random or mixed effects; drift or auto-correlation).*
Effect(s) tested	*Define precise effect in terms of the task or stimulus conditions instead of psychological concepts and indicate whether ANOVA or factorial designs were used.*

Specify type of analysis: ☐ Whole brain ☐ ROI-based ☐ Both

Statistic type for inference<br>(See Eklund et al. 2016)	*Specify voxel-wise or cluster-wise and report all relevant parameters for cluster-wise methods.*
Correction	*Describe the type of correction and how it is obtained for multiple comparisons (e.g. FWE, FDR, permutation or Monte Carlo).*

Models & analysis

n/a	Involved in the study
☐	☐ Functional and/or effective connectivity
☐	☐ Graph analysis
☐	☐ Multivariate modeling or predictive analysis

Functional and/or effective connectivity	*Report the measures of dependence used and the model details (e.g. Pearson correlation, partial correlation, mutual information).*
Graph analysis	*Report the dependent variable and connectivity measure, specifying weighted graph or binarized graph, subject- or group-level, and the global and/or node summaries used (e.g. clustering coefficient, efficiency, etc.).*
Multivariate modeling and predictive analysis	*Specify independent variables, features extraction and dimension reduction, model, training and evaluation metrics.*
